# Genetic Dereplication of Multiple *Penicillium expansum* Biosynthetic Gene Clusters Reveals Cryptic Penta-Cyclopeptide Production

**DOI:** 10.1021/acs.jnatprod.5c01531

**Published:** 2026-02-24

**Authors:** Mira Syahfriena Amir Rawa, Benjamin J. Haefner, Grant R. Nickles, Enrique Aguilar-Ramírez, Nancy P. Keller, Justin L. Eagan

**Affiliations:** † Department of Medical Microbiology and Immunology, 5228 University of Wisconsin-Madison, Madison, Wisconsin 53706, United States; ‡ Department of Plant Pathology, University of Wisconsin-Madison, Madison, Wisconsin 53706, United States

## Abstract

Genetic dereplication by targeted deletion of major secondary metabolite biosynthetic pathways is an effective strategy to uncover novel compounds and cryptic biosynthetic gene clusters (BGCs). In this study, we engineered a “flatline” strain of *Penicillium expansum* by knocking out key biosynthetic genes responsible for producing citrinin, patulin, roquefortine C, andrastin A, and communesins. Comparative metabolomic profiles between the flatline and wild-type strains revealed a marked increase in the production of five-residue cyclopeptides featuring the nonproteinogenic amino acid pipecolic acid in the flatline strain when cultivated on a rice medium. Further analysis led to the identification of known cyclopeptide MBJ-0110 and its new analogues assigned as 3-hydroxy-MBJ-0110 (**1**) and Val-MBJ-0110 (**2**). Their associated BGC was predicted using antiSMASH, whereby deletion of the nonribosomal peptide synthetase (NRPS) *mbjA* abolished the production of these cyclopeptides, confirming the requirement of *mbjA* as the core biosynthetic gene. A phylogenetic search showed a high level of conservation of MbjA across 19 species, predominately in the taxon Eurotiomycetes. By linking biosynthetic genes to these rare cyclopeptides, our study provides support for the genetic dereplication strategy as an effective means to uncover cryptic fungal secondary metabolism.

## Graphical abstract

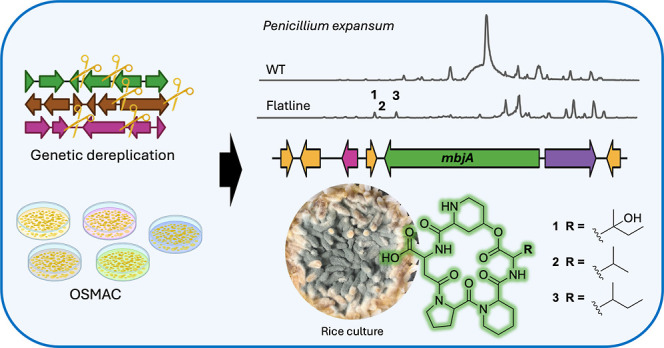

Filamentous fungi are prolific producers of structurally diverse and bioactive secondary metabolites (SMs), many of which are applied in pharmaceutical, agricultural, and food industries.^1–3^ Genome mining and biosynthetic gene cluster (BGC) prediction efforts have identified plentiful, uncharacterized SM potential.^4,5^ A major challenge, however, is inducing BGCs that remain cryptic or silent under standard laboratory conditions. Targeted approaches to induce a cryptic BGC include genetic manipulations or heterologous gene expression, which are effective methods, particularly when an in-cluster transcription factor can be induced.^6^ Untargeted approaches often involve changing culture conditions, such as the one-strain-many-compounds (OSMAC) approach^7^ or coculturing with another microorganism.^8^

Genetic dereplication is another effective strategy for activating cryptic pathways in both targeted and untargeted manner. Dereplication is achieved by deleting the production of major SMs within a single genetic background to create a relatively “flat” metabolomic profile. This provides an opportunity to detect otherwise masked or minor compounds, which has been a successful strategy in several fungi (Table 1). Chiang et al. (2016) pioneered this approach by creating the *Aspergillus nidulans* LO8030 strain in which deletion of eight major BGCs facilitated the discovery of the cryptic lipopeptide aspercryptin.^9^ The LO8030 strain has become an important chassis platform for heterologous expression, leading to the discoveries of tetradeoxy echinocandins,^10^ novel xenoacremone analogues,^11^ and naringenin.^12^ Dereplicated chassis strains developed in *Penicillium crustosum* and *Penicillium rubens* highlight the versatility of this strategy.^13,14^ For example, by eliminating the formation of the reactive ortho-quinone methide in *P. crustosum*, a cleaner metabolic background was generated that prevented nonspecific conversions and enhanced the detection of novel heterologous products.^13^ Beyond simplifying the SM profile for improved detection of cryptic BGCs and compounds, dereplication may also alleviate competition for shared precursors as seen in *P. rubens*, which showed elevated aromatic amino acid levels.^14^

**Table 1:** Genetic Dereplicated Strains Developed from Previous Reports and This Study

no.	fungal platform	deleted BGC/gene	new metabolite	endogenous/heterologous gene/BGC
1	*Aspergillus nidulans* (strain LO8030)	i. sterigmatocystin cluster	aspercryptin	endogenous *atn* BGC^9^
		ii. emericellamide cluster		
		iii. asperfuranone cluster		
		iv. monodictyphenone cluster		
		v. terrequinone cluster		
		vi. austinol parts 1 and 2 cluster		
		vii. F9775 cluster		
		viii. asperthecin cluster		
2	*A. nidulans* (strain LO8030)	see no. 1	tetradeoxy echinocandins	heterologous ecd BGC from *Emericella rugulosa* NRRL 11440 ^10^
3	*A. nidulans* (strain LO8030)	see no. 1	tyrosine-decahydrofluorene analogues: xenoacremones	heterologous *xen* BGC from *Xenoacremonium sinensis* ML-31 ^11^
4	*A. nidulans* (strain LO11098 with multimarkers)	i. sterigmatocystin cluster	sartorypyrones	heterologous *spy* BGC from *Aspergillus fumigatus* ^16^
		ii. emericellamide cluster		
		iii. asperfuranone cluster		
		iv. monodictyphenone cluster		
		v. terrequinone cluster		
		vi. austinol parts 1 and 2 cluster		
		vii. F9775 cluster		
		viii. asperthecin cluster		
5	*A. nidulans* (strain LO11945 with AtbiA-gpdA(p)alcR)	i. sterigmatocystin cluster	tryptophan-containing diketopiperazines: homomorphins A–F	heterologous dimethylallyltryptophan synthases *ahm* BGC from *Aspergillus homomorphus* CBS 101889 ^17^
		ii. emericellamide cluster		
		iii. asperfuranone cluster		
		iv. monodictyphenone cluster		
		v. terrequinone cluster		
		vi. austinol parts 1 and 2 cluster		
		vii. F9775 cluster		
		viii. asperthecin cluster		
6	*A. nidulans* (strain A1145 Δ*ST* Δ*EM*)	i. sterigmatocystin synthase, Δ*stcA*	zaragozic acid A and its intermediate, tricarboxylic acid-containing product	heterologous *clz* BGC from *Curvularia lunata* ^18^
		ii. emericellamide synthase, Δ*easA*		
7	*Penicillium rubens* (strain DS68530)	i. penicillin cluster	decumbenone A–C	heterologous calbistrin BGC from *Penicillium decumbens* ^14^
		ii. roquefortine cluster		
		iii. chrysogine cluster		
		iv. fungisporin cluster		
8	*Penicillium crustosum* (strain PRB-2)	i. terrestric acid synthase, Δ*traA*	annullatin	heterologous *anu* BGC from *Penicillium roqueforti* ^13^
		ii. clavatol, hydroxyclavatol ortho-quinone methide synthase, Δ*claF*		
9	*Trichoderma hypoxylon* Δ*thtri*5	i. trichothecene synthase, Δ*thtri*5	polycyclic lactones tricholactones A and B	endogenous (BGC not characterized)^19^
10	*T. hypoxylon* Δ*thtri*5	i. trichothecene synthase, Δ*thtri*5	sesquiterpenes tricinoloniol acids A–C	endogenous *traA* synthase^20^
11	*Pestalotiopsis fici*	i. pesthetic acid synthase, *pfptaA*	pestaloficiol X	endogenous *iac* BGC^21^
		ii. histone methyltransferase gene, *pfcclA*		
		iii. histone deacetylase gene, *fhdaA*		
12	*Penicillium expansum* (strain TJLE34)	i. citrinin transcription factor and synthase, Δ*ctnA* & Δ*citS*	5-mer cyclopeptide: 3-hydroxy-MBJ-0110 and Val-MBJ-0110	endogenous *mbjA* synthase (this paper)
		ii. patulin transcription factor, Δ*patL*		
		iii. roquefortine synthetase, Δ*roqA*		
		iv. communesin cluster, Δ*cns*		
		v. Pex2_030390 unknown synthase		
		vi. andrastin synthase, Δ*adrD*		

Building on this concept, we developed a dereplicated *Penicillium expansum* strain, designated the “flatline” strain, through targeted deletions of five major SM pathways: citrinin, patulin, roquefortine C, andrastin A, and communesins. As one of the most extensively studied plant pathogens, *P. expansum* poses a threat to human health through its biosynthesis of mycotoxins, especially patulin, making the research of its SMs crucial for understanding its pathogenicity.^15^ In this study, the deletion of these major SMs resulted in a markedly reduced metabolomic complexity under laboratory conditions. In addition, cultivation of the strain through the OSMAC technique led to the discovery of uncharacterized five-residue cyclopeptides containing the nonproteogenic amino acid pipecolic acid (Pip), assigned as 3-hydroxy-MBJ-0110 (**1**) and Val-MBJ-0110 (**2**), along with their known analogue, MBJ-0110 (**3**), described here for the first time from *P. expansum*. We elucidated the nonribosomal peptide synthetase (NRPS) BGC responsible for biosynthesis. This finding represents the first genetic assignment of a BGC to cyclopeptides **1**–**3** production, providing a direct link between genotype and chemical phenotype for this rare cyclopeptide family and setting the stage for future biosynthetic engineering efforts.
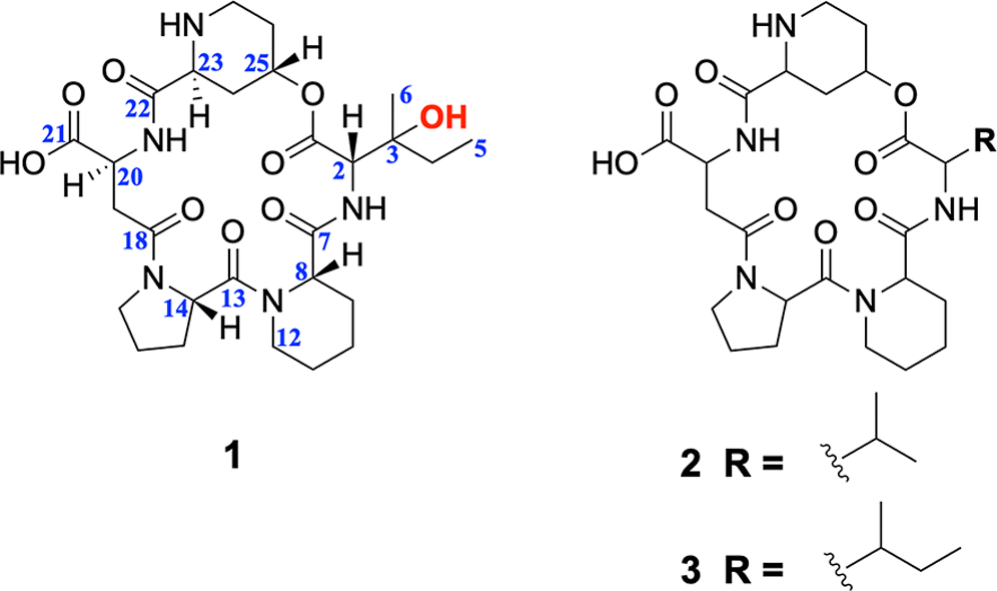


## Results and Discussion

### Genetic Dereplication Results in the Loss of Five Major SM Biosynthetic Pathways in *P. expansum*

The first two SM pathways we targeted were patulin and citrinin, both major mycotoxin compounds produced by *P. expansum* (strain Pe-21).^15,22^ We deleted in-cluster transcription factors *patL* and *ctnA* to abolish patulin and citrinin production, respectively. Our liquid chromatography tandem mass spectrometry (LC–MS/MS) analysis of patulin confirmed loss in Δ*patL* backgrounds.^23^ Under the same culture conditions of 14 days on potato dextrose agar (PDA), citrinin was not detected in the double deletion strain TJLE4 (Table S1 and Figure S1A). However, an earlier time point of 10 days on PDA revealed low levels of citrinin in TJLE4 (Figure S1B). At this time point, patulin was still not detected (Figure S1C). A time course experiment with wild-type (WT) suggested that citrinin detection is dependent on the culture time (Figure S1D). This supports a study that reported oscillatory kinetics of citrinin production on PDA over time.^22^ Our lab previously reported the promiscuity of PeXanC to induce citrinin production *in trans*; however, deletion of *ctnA* was sufficient to prevent PeXanC-mediated activation after 14 days on PDA.^24^ Here, our data suggest that more incremental time points are needed to parse this regulatory crosstalk and/or another method by which the citrinin BGC is induced. To ensure complete loss of citrinin, we deleted the citrinin synthase *citS* (Figure S1E).^24^ Going forward, we deleted the synthetase *roqA*, necessary for roquefortine C production^25^ and the synthase *adrD*, necessary for andrastin production.^26^ For deleting the production of communesins, we opted to delete the entire BGC to avoid potential shunt products similar to those observed by Lin et al. (2015) who elucidated the biosynthetic pathway in *P. expansum*.^27^ Resulting extracts from the final flatline strain, TJLE34, grown on PDA showed that citrinin, patulin, roquefortine C, andrastin A, and communesins were not produced from the five disabled BGCs, as determined by LC–MS/MS profiles (Figure S2). Additionally, the production of analogues roquefortine D and andrastins B–F was also abolished (Table S2).

### OSMAC Approach Unveils Increased Production of Unique Peaks in the Flatline Strain

The OSMAC approach is a highly effective method in facilitating SM discovery by leveraging varied cultivation conditions to increase natural product titer and chemical diversity.^7^ For instance, fermentation of *Penicillium* sp. (strain HLLG-122) yielded nine new highly oxygenated meroterpenoids when incorporating different media compositions (solid rice and potato dextrose broth (PDB) liquid media).^28^ In this study, we assessed the full SM potential of the flatline strain by growing both the WT and flatline strains on 15 different solid media (Table S3 and Figure S3). LC–MS/MS analysis of extracts showed the production of diverse SMs in the flatline strain especially when grown on grain-based media such as oats and rice (Figure S4). For example, the abundance of tetracyclic sesquiterpene lactones, expansolides A and B (*m*/*z* 307.1540),^29,30^ in the flatline strain were increased by almost 2-fold when cultivated on rice compared to WT (Figures 1A and S4). Subsequently, global natural products social (GNPS) molecular networking of the positive ion mode MS/MS data highlighted unique metabolites of the flatline extracts from the rice culture compared to those from WT (Figure 1B). Particularly, a molecular family comprising 16 nodes (i.e., 16 putatively related SMs) included three metabolites with *m*/*z* values of 580.2964 (**1**), 550.2860 (**2**), and 564.3014 (**3**), which could not be dereplicated in GNPS due to the absence of the corresponding MS/MS reference spectra (Figure 1B). Fragmentation analysis suggested that they are related peptides containing an uncommon amino acid Pip, highlighting their novelty. Given their abundance in the rice culture (Figure S5) and unique structural features, these metabolites were therefore targeted for isolation and BGC characterization.

**Figure 1:**
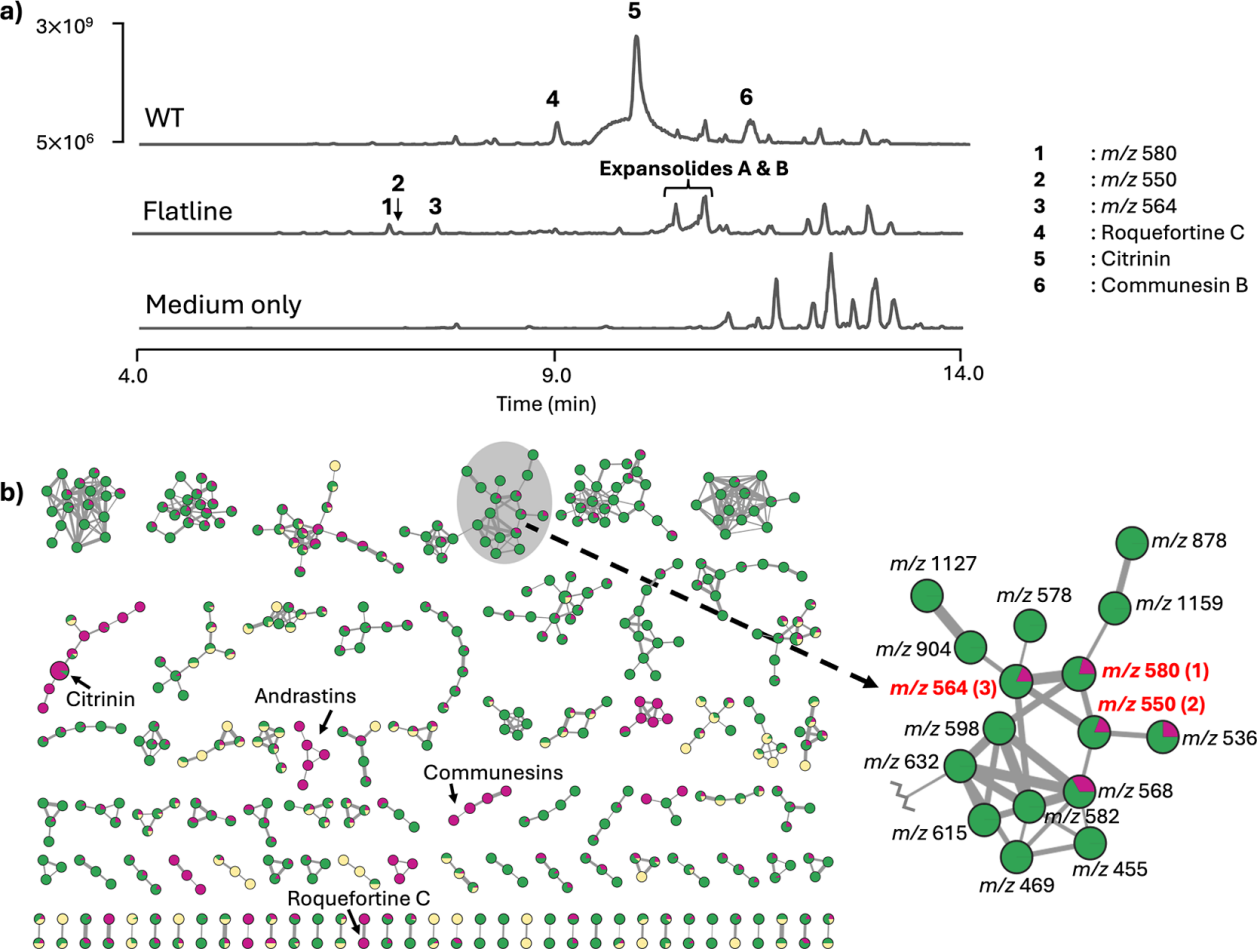
Metabolite profiling of the flatline strain based on reverse-phase LC–MS data analysis. (a) TICs in positive ion mode of *P. expansum* WT and flatline strains cultivated on rice medium. Deleted key biosynthetic genes abolished the metabolite peaks of five major SMs including citrinin, roquefortine C, and communesins. The peak intensity of TIC was standardized to 5 × 10^6^ to 3 × 10^9^, at a retention time window of 4–14 min (b) Partial classical GNPS molecular network (+ ion mode LC–MS/MS data) of *P. expansum* strains cultivated on rice medium (green = flatline strain TJLE 34.1, purple = WT strain TJT 14.1, yellow = rice medium only). SMs **1**–**3** were upregulated in the flatline strain. SMs corresponding to deleted biosynthetic genes were observed in the clusters within the WT strain only.

### Isolation and Structure Elucidation Led to the Discovery of New Cyclopeptides 1 and 2

Compound **1** was obtained as a colorless amorphous powder, and its molecular formula was established as C_27_H_41_N_5_O_9_ by HR–electrospray ionization (ESI)–MS (*m*/*z* 580.2964 [M + H]^+^, calculated for C_27_H_42_N_5_O_9_ *m*/*z* 580.2983). Its peptidic nature was deduced from its MS/MS fragmentation pattern, which showed the loss of residue masses in the fragment ions (Figure S7). The molecular formula of compound **3** was established as C_27_H_41_N_5_O_8_ by HR–ESI–MS (*m*/*z* 564.3014 [M + H]^+^, calculated for C_27_H_42_N_5_O_8_ *m*/*z* 564.3033). A search for the accurate mass (Figure S8) and comparison of nuclear magnetic resonance (NMR) data (Figures S9–S11 and Table S4) with the reported paper^31^ confirmed the identification of **3** as MBJ-0110, a Pip-containing cyclopeptide isolated from *Penicillium* sp. (strain f25267).

The ^1^H NMR spectrum of **1** showed high-fielded chemical shift values of a triplet methyl and a singlet methyl signals resonating at 0.91 and 1.23 ppm, respectively (Figure S12). Six methine protons resonating at 4.12–5.17 ppm represent α-protons of amino acid moieties, while 12 methylene protons observed at 1.50–3.97 ppm were indicative of β- and aliphatic protons. The ^13^C NMR spectrum of **1** revealed 27 signals (Figure S13), which was consistent with the chemical formula confirmed by HR–ESI–MS. The ^13^C DEPT experiment further validated the presence of 2 methyl, 12 methylene, 6 methine, and 7 quaternary carbons (Figure S14). Carbon resonances of a methine carbon and a quaternary carbon observed at 69.6 and 73.5 ppm, respectively, signified the occurrence of an electronegative atom attached to these carbons. Table 2 summarizes the ^13^C and ^1^H NMR spectroscopic data for **1**.

**Table 2:** ^1^H and ^13^C NMR spectroscopic data of **1** in D_2_O^a^

position	type	δ_C_	δ_H_, mult. (*J* in Hz)
1	C	169.3	-
2	CH	61.4	4.40, s
3	C	73.5	-
4	CH_2_	33.4	1.57, q (7.7)
5	CH_3_	8.5	0.91, t (7.5)
6	CH_3_	23.7	1.23, s
7	C	174.6	-
8	CH	55.3	5.13, dd (9.0, 2.8)
9	CH_2_	24.5^a^	1.75 overlap 2.24, overlap
10	CH_2_	20.2	1.74, overlap 1.50, overlap
11	CH_2_	24.5^a^	1.74,overlap 2.22, overlap
12	CH_2_	44.7	3.13, overlap 3.97, d (13.4)
13	C	174.9	-
14	CH	59.5	5.02, dd (9.2, 3.7)
15	CH_2_	29.8	2.00, overlap 2.51, m
16	CH_2_	25.4	2.00, overlap
17	CH_2_	50.4	3.64, dt (10.7, 5.9) 3.75, dt (11.2, 7.2)
18	C	172.1	-
19	CH_2_	37.1	2.89, d (13.1) 3.18, overlap
20	CH	51.9	4.72, d (7.7)
21	C	177.1	-
22	C	169.3	-
23	CH	54.9	4.12, dd (13.0, 3.0)
24	CH_2_	32.0	2.17, overlap 2.65, d (15.0)
25	CH	69.6	5.17, br. s
26	CH_2_	25.6	2.04, overlap 2.39, d (15.7)
27	CH_2_	39.9	3.23, overlap 3.46, dd (13.4, 4.7)

a The carbon chemical shifts are interchangeable.

The direct correlations between carbons and their attached protons were determined based on the heteronuclear single-quantum coherence experiment (Figure S15). The ^1^H spin systems and the long-range ^1^H–^13^C couplings between α-methine protons and their corresponding amide carbonyl carbons were elucidated using homonuclear correlation spectroscopy (COSY) and heteronuclear multiple bond correlation (HMBC) spectra, respectively (Figures 2, S16, and S17). The two-dimensional (2D) NMR data revealed the presence of Pip, proline (Pro), and aspartic acid (Asp). Additionally, the occurrence of a γ-hydroxypipecolic acid moiety (hydroxy-Pip) similar to MBJ-0110 ^31^ was demonstrated based on the HMBC correlations of a nitrogen-bearing methylene proton H-27 (3.23 and 3.46 ppm) and an aliphatic methylene proton H-24 (2.17 and 2.65 ppm) with an oxymethine carbon C-25 (69.6 ppm), and an α-methine proton H-23 (4.12 ppm) with H-27 and an amide carbonyl carbon C-22 (169.3 ppm).

The presence of a β-hydroxyisoleucine (hydroxy-Ile) residue in compound **1** was indicated by HMBC NMR correlations (Figures 2 and S18) of the hydroxylated quaternary carbon C-3 (73.5 ppm) with an α-methine proton H-2 (4.40 ppm), two methyl protons H-5 and H-6 (0.91 and 1.23 ppm, respectively), and a methylene proton H-4 (1.57 ppm). The observed downfield chemical shift of C-3 suggests deshielding of the carbon nucleus by an electronegative atom, consistent with the presence of an attached oxygen atom. The singlet observed for the methyl proton at C-6 supported the presence of the hydroxy group attached to the β-quaternary carbon, while the triplet multiplicity of the methyl proton at C-5 suggested its coupling with a neighboring methylene proton (H-4), a characteristic of an Ile. These data confirmed the structure of **1** as a new derivative of MBJ-0110 containing a hydroxylated Ile moiety at C-3, designed as 3-hydroxy-MBJ-0110.

**Figure 2:**
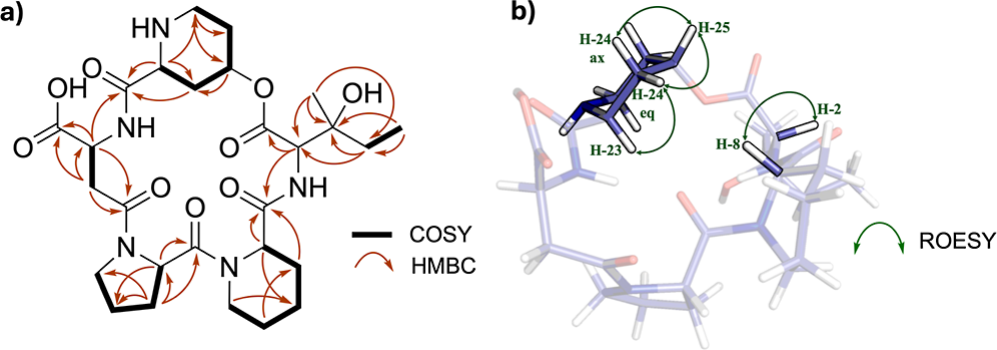
2D correlations of **1**. (a) Key COSY and HMBC correlations of **1**. (b) 3D model of **1** with ROESY correlations supporting the stereochemistry assignment.

The HMBC correlations verified the partial amino acid sequence of **1**, indicating that Asp is adjacent to hydroxy-Pip, and hydroxy-Ile is positioned next to Pip (Figure 2). Treatment of **1** with 0.1 N NaOH overnight at room temperature (Figure 3A), followed by ESI–MS/MS analysis established the molecular formula of the alkaline hydrolysate as C_27_H_43_N_5_O_10_ (HR-ESI–MS: [M + H]^+^ *m*/*z* 598.3087, calculated for C_27_H_44_N_5_O_10_ 598.3088). The ESI–MS/MS data showed major fragment ions (*m*/*z* 185.0926, 243.0980, 324.1560, 340.1508, and 451.2191) that supported the proposed structure (Figure 3B). Basic hydrolysis cleaved the lactone ring of **1**, leading to a linear peptide having the amino acid sequence: hydroxy-Ile–Pip–Pro–Asp–hydroxy-Pip.

**Figure 3:**
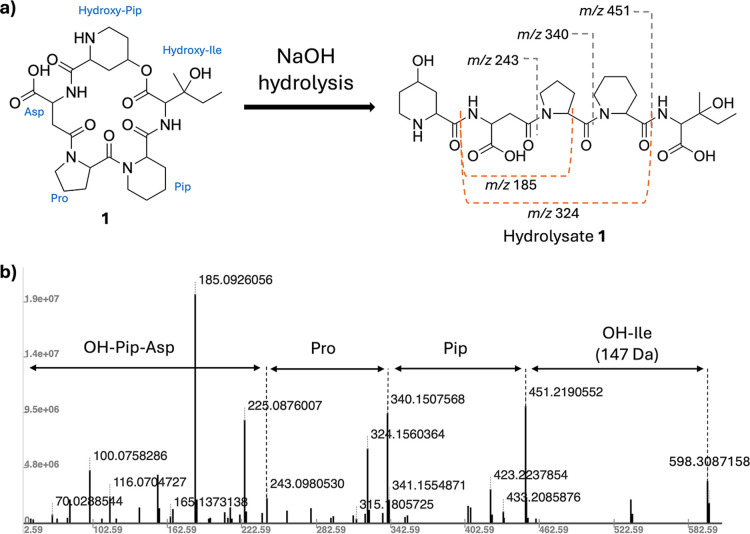
Structural information on **1**. (a) NaOH hydrolysis of **1**. b) ESI–MS/MS fragmentation ions of hydrolysate **1** showing the sequence of amino acids.

The absolute configurations of the amino acid residues in compound **1** were determined as L-Pro, L-Pip, and L-Asp using the advanced Marfey’s method,^32^ consistent with the reported absolute configuration of MBJ-0110 (Figure S19 and Table S4). As Pip possesses an *S* configuration at its α-carbon (C-8), biosynthetic logic suggests that C-23 should bear the same configuration, as no epimerase domain is detected in the NRPS modules. Consistent with this assignment, H-23 (4.12 ppm) is proposed to be α-oriented and shows axial–axial coupling (*J* = 13.0 Hz) with H-24 β-axial (2.17 ppm) as well as axial–equatorial coupling (*J* = 3.0 Hz) with H-24 α-equatorial (2.65 ppm) (Figure 2). In support of this, H-23 displays a rotating frame Overhauser effect spectroscopy (ROESY) correlation with H-24 α-equatorial but not with H-24 β-axial, indicating that H-23 and H-24 β-axial are located on opposite faces (Figure 2). Using analogous reasoning, H-25 (δ 5.17 ppm) is proposed to be β-oriented (*S* configuration), as it exhibits ROESY correlations with both axial and equatorial H-24 protons.

The observed ROESY correlation between H-8 (5.13 ppm) and H-2 (4.40 ppm) indicates that H-2 is β-oriented, allowing for the assignment of an *S* configuration at C-2 (Figures 2 and S20). However, the configuration at C-3 could not be determined solely on the basis of the ROESY correlations between H-2 and H-6, as the H-2/H-6 distance is similar for the peptide in either the 3S* or 3R* configuration (data not shown).

The molecular networking analysis (Figure 1B) highlighted compound **2** as a related cyclopeptide of **1** and MBJ-0110. The molecular formula of **2** was established as C_26_H_39_N_5_O_8_ by HR–ESI–MS (*m*/*z* 550.2860 [M + H]^+^, calculated for C_26_H_40_N_5_O_8_ *m*/*z* 550.2877), consistent with the loss of one CH_2_ group compared to MBJ-0110 (Figure S21). Although isolation of **2** could not be completed due to the low yield, its fragmentation pattern shows product ions at *m*/*z* 433.2097, 322.1411, and 225.0876, corresponding to the sequential fragmentation of valine (Val), Pip, and Pro (Figure S21). These data suggest that this cyclopeptide is a new analogue of MBJ-0110 in which Ile is replaced by Val and was therefore assigned as Val-MBJ-0110. This discovery is supported by the biosynthetic logic of a cyclopeptide group called destruxins from *Metarhizium* spp, where its adenylation (A) domains exhibit similar substrate flexibility to generate structurally related analogues.^33^

Compounds **1**–**3** are members of a cyclic depsipeptide group comprising at least one lactone bond in the core ring. Cyclic depsipeptides from fungi are structurally diverse, ranging from 3 up to 13 residues.^34^ Among the Pip-bearing cyclopeptides, petrosifungins A–B and JBIR 113–115 originate from *Penicillium* spp.^35,36^ Compounds **1**–**3** are unique in that they contain a Pip with a hydroxyl group that forms a lactone bond with the C-terminal carboxyl of its neighboring amino acid rather than the more common amide bond to create a peptide cyclization. Compound **1** represents, to the best of our knowledge, the first example of a 3-hydroxylated Ile incorporated into a fungal peptide, although hydroxy-Ile formation was previously documented in bacteria such as *Bacillus thuringiensis* (strain 2 × 10^2^).^37,38^ Moreover, such hydroxylation is rare at the C-3 position, with C-4 being a more common site,^37,38^ further underscoring the novelty and chemical diversity of this fungal compound. This mechanism of using nonstandard amino acids adds another biosynthetic insight into how such diversity arises.

### NRPS MbjA Is Responsible for the Production of Cyclopeptides **1**–**3**

Next, we were interested in identifying the BGC producing these cyclopeptides. Given that cyclopeptides **1**–**3** are composed of five residues, we hypothesized that an NRPS with five A domains would be responsible for its synthesis. AntiSMASH (https://fungismash.secondarymetabolites.org/) predicted 21 regions encoding NRPSs within the *P. expansum* genome (Supporting Information data 2).^39^ Further analysis of these 21 regions using PARAS (https://paras.bioinformatics.nl/)^40^ revealed two NRPSs (PEXP_055140 and PEXP_085540) with five and six predicted A domains, respectively (Supporting Information data 2). Considering this prediction, we deleted both NRPS genes using the flatline strain as the background strain, which were then analyzed for their products via LC–MS/MS analysis. The flatline strain containing the deleted PEXP_055140 gene (termed *mbjA*) showed the loss of cyclopeptides **1**–**3**, confirming the requirement of MbjA for the production of these cyclopeptides (Figure 4).

**Figure 4:**
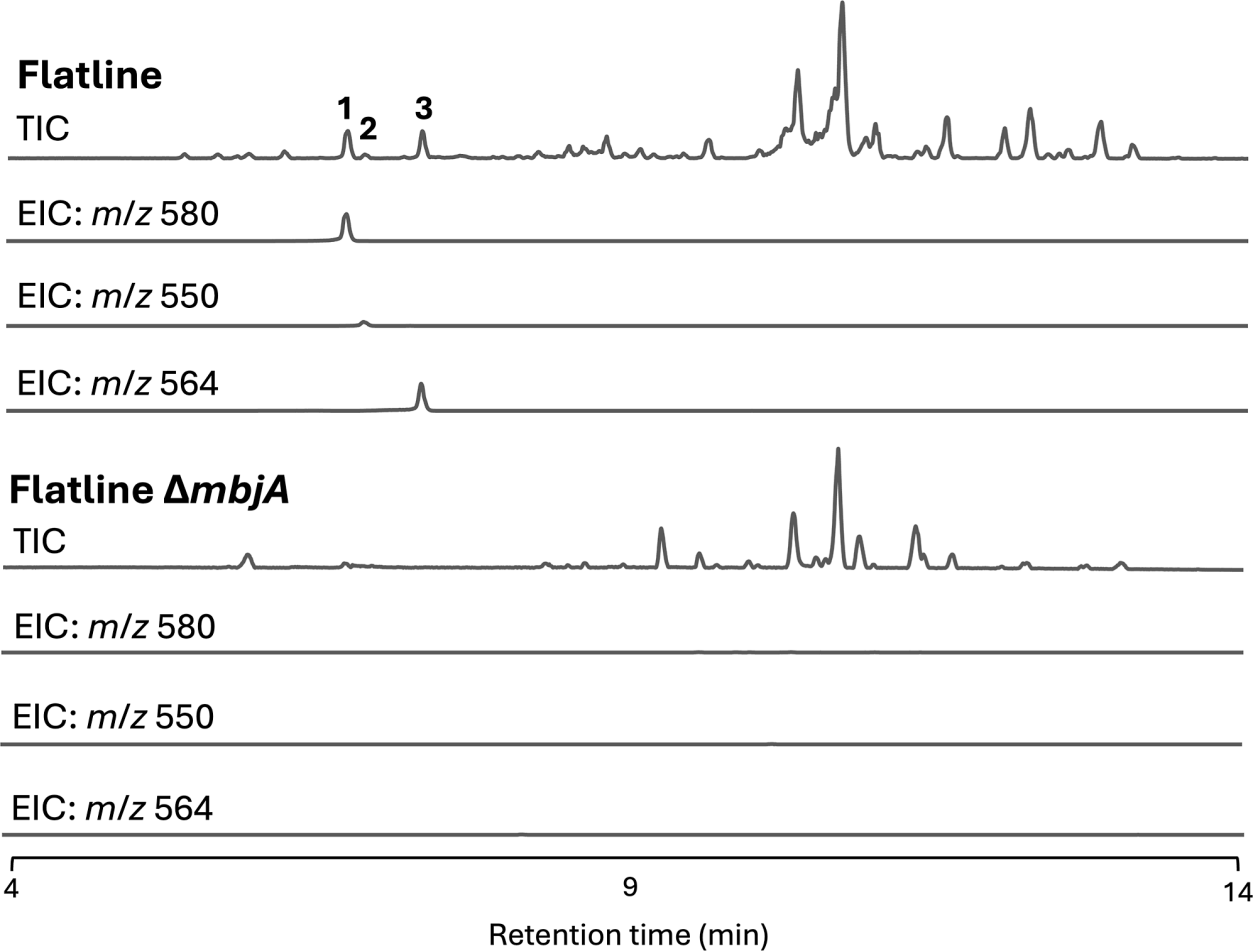
PEXP_055140 (*mbjA*) is responsible for cyclopeptides **1**–**3** production. TICs and EICs (+ ion mode) of the flatline and flatline Δ*mbjA* strains. Deletion of *mbjA* abolished the production of cyclopeptides **1**–**3**.

### MbjA NRPS Is Found across Distant Taxa despite Cluster Specificity to the *Penicillium* Genus

Although the *mbjA*-encoded NRPS is hypothetically sufficient to biosynthesize the peptide backbones of **1**–**3**, the genomic origin of the necessary nonproteinogenic precursors (e.g., l-Pip) remains unclear. Specifically, it is unknown if the auxiliary enzymes required to synthesize these precursors are colocalized within the *mbj* BGC or encoded *in trans*. To address this, we investigated the evolutionary conservation of this pathway under two distinct assumptions. First, assuming the intact *mbj* BGC is required for biosynthesis, we searched for conserved loci in other species using cBlaster.^41^ Second, assuming *mbjA* functions autonomously or cooperates with genomically distal tailoring enzymes, we performed a broad BLASTp search followed by synthaser^42^ and PARAS^40^ analyses to identify five-module MbjA homologues that were independent of the predicted cluster.

Our analysis of the full *mbj* locus revealed that while many *Penicillium* spp. possess a homologue of the BGC, it is not ubiquitous within the genus (Figure 5A). Among the *Penicillium* species containing the cluster, genomic synteny was high, with the majority of enzymatic genes being conserved (Figure 5A). In contrast, our search for the NRPS alone identified 30 unique homologues across 19 different species (Figure 5B). These homologues were predominantly found in the Eurotiomycetes (specifically *Aspergillus*, *Penicillium*, and *Talaromyces*) but were also identified in select Sordariomycetes (*Trichoderma* spp.) and a single Dothideomycete (*Lasiodiplodia* *theobromae*). Notably, every genome containing an *mbjA* homologue possessed exactly one copy. Interestingly, the amino acid sequence similarity between homologues from the *Trichoderma* and *Penicillium* genera was identical to that observed between the much taxonomically closer *Aspergillus* and *Penicillium* genera.

**Figure 5:**
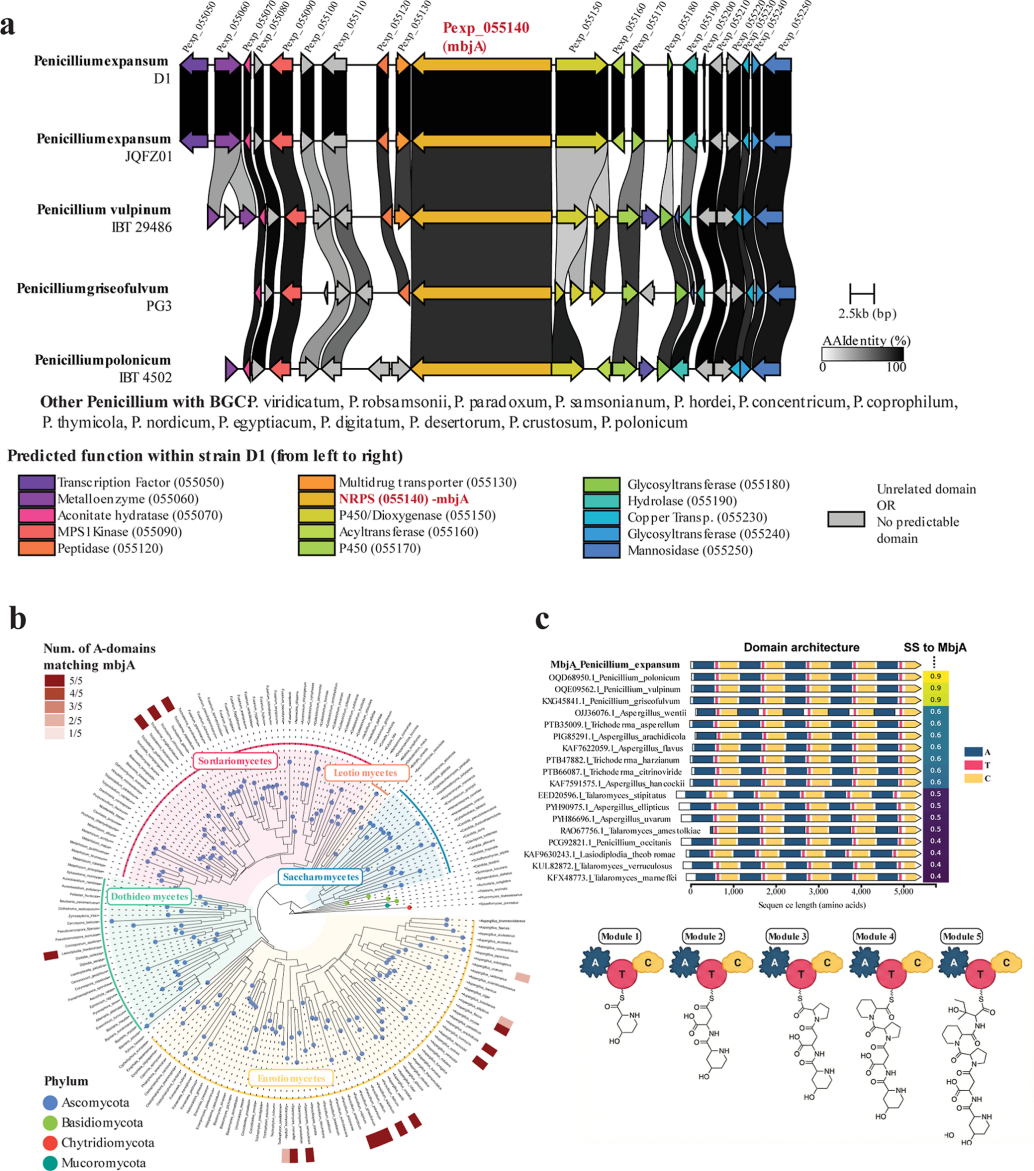
Distribution of the *mbj* BGC and *mbjA* encoding NRPS. (a) Synteny alignment of the *mbj* BGC in *P. expansum* Pe-21 (D1 on NCBI) with homologous loci in five other *Penicillium* species. Predicted functions for each gene are listed, along with additional *Penicillium* spp. containing homologous *mbj* loci. (b) Phylogenetic distribution of species encoding MbjA homologues with exactly five adenylation (A) domains. Tree tips indicate phylum, and major taxonomic classes are highlighted. Heatmap intensity (white to red) indicates the number of A domains matching the substrate specificity of the *P. expansum* Pe-21 MbjA reference. c) Domain architecture of MbjA compared to homologues identified in distantly related species. The proteins are sorted based on the amino acid sequence similarity relative to MbjA, and the values are displayed to the right of each protein. The proposed function of each MbjA module in the biosynthesis of **1** is illustrated.

To verify that these distant homologues were likely functional orthologues rather than unrelated NRPSs with a similar module count, we predicted A-domain substrate specificity using PARAS (Figure 5C). Of the 30 identified homologues, 20 exhibited an A-domain specificity profile identical to that of the *P. expansum* Pe-21 MbjA reference across all five modules. The remaining homologues showed high conservation, with only minor deviations in predicted specificity (4/5 modules matching in one protein; 3/5 matching in eight proteins).

Collectively, the strong sequence similarity, identical domain architecture, and conserved substrate specificity profiles suggest that these enzymes are biochemically equivalent to MbjA. While the exact evolutionary history of this NRPS requires further phylogenetic reconciliation, the observed distribution allows for several hypotheses. The presence of *mbjA* in distantly related Sordariomycetes and Dothideomycetes, despite its absence in many closer relatives, suggests potential horizontal gene transfer events from the Eurotiomycetes to these lineages.^43^ Alternatively, strong environmental selection for the production of cyclopeptides **1**–**3** could have driven convergent evolution, leading to the independent emergence of structurally similar NRPSs in these distant lineages, though the high degree of sequence identity makes this less probable for the homologues found in the *Trichoderma* genus.^44^ Finally, this distribution could result from the extensive lineage-specific loss of an ancient NRPS gene family that predated the divergence of these fungal classes, although this scenario is less parsimonious given the broad absence of the gene in the majority of Ascomycota.^45^

The structures of cyclopeptides **1**–**3** containing l-Pip suggest a requirement of an enzyme to make this nonproteinogenic amino acid. The AntiSMASH analysis did not predict a cyclodeaminase as one of the neighboring genes to *mbjA* (Figure 5A), which is typically responsible for converting l-lysine to l-Pip (e.g., RapL).^46^ This absence may imply that the incorporation of l-Pip critical to natural products like rapamycin^46^ may rely on a cyclodeaminase encoded outside the core gene cluster. However, *mbjA’*s neighboring gene encoding PEXP_055150 is predicted to be a dioxygenase/2OG-Fe­(II) oxygenase that may be responsible for hydroxylating l-Pip to form hydroxy-l-Pip. This reaction is analogous to those in other fungi like in *Fusarium oxysporum* ^47^ utilizing nonheme iron, Fe­(II), and the cosubstrate 2-oxoglutarate (2OG) to catalyze various reactions, including hydroxylation of *L*-Pip.^47,48^ It is also possible that PEXP_055150 plays a role in the hydroxylation of Ile as seen in compound **1**. Considering the core ring formation of these cyclopeptides, this cyclization could be mediated either by a standard thioesterase (TE) domain^49^ or by a specialized condensation (C) domain integrated within the NRPS assembly line without the need for a terminal TE.^50^ This hypothesis is exemplified by the destruxin NRPS (DtxS1) assembly line. DtxS1 lacks a TE domain at the carboxyl terminus and may instead rely on its terminal C domain containing the catalytic SHAQYDG motif accountable for cyclization.^33^ The occurrence of a linear protodestruxin^51^ nonetheless suggests that cyclization mediated by an external TE domain remains a possible route, illustrating the two cyclization possibilities for the MbjA system as well.

## Conclusion

In summary, by eliminating five dominant metabolite pathways, we created a cleaner chemical profile in *P. expansum* under the standard laboratory conditions. This genetic dereplication strategy, enhanced by the OSMAC approach, enabled the discovery of new cyclopeptide derivatives featuring uncommon amino acids such as hydroxy-Pip and hydroxy-Ile as well as the identification of its core biosynthetic gene, *mbjA* with demonstrated substrate flexibility that underscores the pathway’s potential for chemical diversity. Our findings demonstrate the direct genetic basis for these rare cyclopeptides and open the door for exploring fungal chemistry that would otherwise remain hidden.

## Experimental Section

### General Experimental Procedures

Optical rotations were measured on an Autopol III Automatic Polarimeter (Rudolph Research Analytical, Hackettstown, NJ, USA). Ultraviolet (UV) spectra were recorded on a Gilson 171 DAD detector (Middleton, WI, USA). Infrared (IR) spectra were recorded on a Nicolet 6700 Fourier transform infrared spectrometer (Thermo Electron Scientific Instruments Corporation, Madison, WI USA). NMR spectra were recorded in water-*d*_2_ (D_2_O) solution on a Bruker Avance III HD, 500 MHz instrument (Billerica, MA, USA) equipped with a 5 mm cryoprobe at 500 MHz for ^1^H NMR and 125 MHz for ^13^C NMR. The NMR spectra were processed and baseline-corrected using the MestReNova software. ^1^H chemical shifts were referenced to the residual D_2_O or CD_3_OD solvent, while ^13^C chemical shifts were referenced to the residual CD_3_OD solvent signal for compound **1**, after the acquisition in 100% D_2_O (approximately 10% CD_3_OD was added to enable accurate ^13^C NMR referencing). Chemical shifts were expressed in parts per million (δ). MPLC was accomplished by using a Teledyne ISCO CombiFlash Companion (Teledyne ISCO, NE, USA). Ultra-high-performance liquid chromatography (UHPLC)–HRMS and UHPLC–MS/MS data were acquired using a Thermo Fisher Scientific Vanquish UHPLC system (Waltham, MA, USA) with a Waters XBridge BEH-C18 column (2.1 × 100 mm, 1.7 μm) that was connected to a Thermo Fisher Scientific Q Exactive Hybrid Quadrupole-Orbitrap mass spectrometer (Waltham, MA, USA) operated in positive and/or negative ionization modes using a mass/charge ratio (*m*/*z*) range of 100–1500. Preparative HPLC was conducted using a Gilson 332 pump and a Gilson 171 DAD detector (Middleton, WI, USA) with an XBridge Prep C18 OBD column (5 μm, 19 × 250 mm). All solvents used were spectroscopic grade.

### Strain Construction and Maintenance

The *P. expansum* apple isolate Pe-21 (also named D1 on NCBI)^52^ served as the progenitor strain, with TJT14.1 (Δ*ku*70:six-site) used as the WT reference in this study.^53^ To generate the flatline strain, we employed a recyclable hygromycin-resistance cassette^54^ as a selectable marker for homologous recombination, enabling targeted deletion of biosynthetic genes responsible for the production of citrinin, patulin, roquefortine C, communesins, and andrastins. All strains used in this study, including intermediate mutants generated during the stepwise construction process, are listed in Table S2. Notably, an attempt to disrupt chaetoglobosin biosynthesis by deleting *PEX*2_030390 inadvertently removed an unrelated NRPS-PKS gene. All strains were confirmed with Southern blot or PCR assays (Figure S22).

To excise the hygromycin-resistance cassette, transformants were cultured for two successive passages on xylose minimal medium (XMM; glucose minimal medium [GMM] with 2% (w/v) xylose replacing glucose). This medium induces the expression of a β-recombinase, driven by the xylose-inducible *xylP* promoter from *Penicillium chrysogenum*, which targets flanking six-site recombination sequences and facilitates cassette excision from the genome.^54^ Successful hygromycin cassette integrations were verified by Southern blotting, while cassette excisions were confirmed via PCR (Figure S22). For long-term preservation, conidial spores from each strain were harvested from GMM plates using 0.01% Tween-20 and stored as 25% (final concentration) glycerol stocks at −80 °C. Importantly, in the final flatline strains (TJLE34.1/2/3), the hygromycin-resistance cassette integrated at the *adrD* locus could not be excised despite multiple passage attempts on XMM. All sibling isolates failed to recycle the marker, indicating stable integration at this locus.

Double-joint PCR was used to generate gene-deletion constructs, following the method described by Yu et al., 2004.^55^ For each NRPS (PEXP_055140 and PEXP_085540), 1.5 kb regions upstream and downstream of the open reading frame as well as the selectable marker were amplified using Pfu DNA polymerase (Agilent, Santa Clara, CA, USA). Amplification primers included 23 bp (upstream) and 26 bp (downstream) overhangs complementary to the selectable marker sequence. An excisable hygromycin resistance cassette from plasmid pSK529 ^54^ was used as the selectable marker. Following amplification, each flank was gel-purified by using the QIAquick PCR Purification Kit (Qiagen, Hilden, Germany). The purified fragments were combined with the selectable marker fragment at a 1:5:1 ratio (5′ flank: marker: 3′ flank) for fusion PCR. The assembled construct was then amplified with nested primers using the Long Template Expand PCR System (Roche, Indianapolis, IN, USA), and the final PCR product was purified using a G-50 column (Cytiva, Marlborough, MA, USA). Primer sequences used for construct generation are listed in Table S6.

NRPS deletion strains were generated through homologous recombination using protoplast-mediated transformation, as previously described.^56^ The parental strain MJLE6.1 served as the transformation background. To generate protoplasts, approximately 10^9^ spores were cultured in liquid GMM supplemented with 0.1% (w/v) yeast extract at 25 °C with agitation at 250 rpm until germ tube formation was evident. Germlings were pelleted by centrifugation, rinsed with sterile water, and resuspended in 30 mL of protoplasting solution (Osmotic Medium containing 0.06 g of Yatalase and 0.1 g of Lysing Enzymes from *Trichoderma*). This suspension was incubated overnight at 25 °C at 100 rpm to facilitate cell wall digestion. Protoplasts were collected by centrifugation with 8 mL of Trapping Buffer at 3750 rpm for 15 min at 15 °C, washed with STC buffer (1.2 M sorbitol, 10 mM CaCl_2_, 10 mM Tris–HCl, pH 7.5), and resuspended in 500 μL of the same buffer. For transformation, 100 μL of protoplast suspension was combined with 25 μL of the gene-deletion cassette in a final volume of 200 μL STC buffer and incubated on ice for 50 min. Following this, 1.25 mL of 60% PEG 4000 solution was added, and the mixture was incubated at room temperature for 15 min. Afterward, 5 mL of STC buffer was added, and the mixture was placed on ice for 30 min. To facilitate recovery, 10 mL of SMM medium was added, and cultures were incubated overnight at 25 °C with shaking at 250 rpm. The following morning, the recovered germlings were mixed with molten SMM top agar supplemented with hygromycin (100 μg/mL; final agar concentration 0.7%), and the mixture was poured over SMM plates containing hygromycin (100 μg/mL). Plates were incubated at 25 °C for approximately 5 days until colonies emerged. Transformants were transferred to fresh selective media, subjected to DNA extraction, and verified by PCR using primers binding outside the homologous flanking regions together with marker-specific primers to confirm correct integration.

### Culture Methods for SM Extractions

For all experiments, the inoculum size was standardized to 500,000 spores, corresponding to 10 μL of a 5 × 10^7^ spores/mL suspension. For solid media, including plate- and grain-based cultures, spores were point-inoculated at the center of the medium. In liquid culture experiments using PDB, 10 μL of the spore suspension was added directly to 3 mL of media, and the mixture was gently mixed. All media formulations used in this study are detailed in Table S4. Cultures were incubated at 25 °C in the darkness.

### SM Extraction and LC–MS/MS Analysis

Following the incubation period, the entire culture (agar medium and fungal colony) was harvested and transferred to 50 mL conical tubes and then frozen at 80 °C for a minimum of 1 h to overnight. Samples were subsequently lyophilized for approximately 1 week. Once thoroughly dried, samples were homogenized by manual grinding with a glass rod, followed by extraction in 20 mL of methanol under shaking conditions (25 °C and 250 rpm) overnight. The initial extract was decanted into a fresh conical tube, and a second methanol extraction was performed on the residual material. Combined extracts (∼40 mL per sample) were allowed to dry completely under ambient conditions in a fume hood. Dried residues were reconstituted in methanol to a final concentration of 1 mg/mL for chromatographic analysis. For Figure S1A–C, ethyl acetate extractions were performed as previously described.^23^

UHPLC–HRMS and UHPLC–MS/MS analyses were performed with a 10 μL injection volume and a flow rate of 0.2 mL/min. The 20 min linear gradient method of water (H_2_O)/acetonitrile (MeCN) with 0.05% formic acid was used: initial hold at 5% organic phase for 2.5 min, increase to 98% over 10.5 min, hold at 98% for 4 min, decrease to 5% over 0.5 min, and final hold at 5% for 2.5 min.

### Feature Detection and Characterization

LC–MS/MS raw data files were converted to the centroided.mzXML format using RawConverter (v1.2.0.1, The Scripps Research Institute). Data acquired in positive and negative ionization modes were processed independently. Targeted feature detection and quantification were performed using Maven (v2.0.3), where specific *m*/*z* values of interest were extracted and intensity values were subsequently visualized using GraphPad Prism (v10). Total ion chromatograms (TICs) and extracted ion chromatograms (EICs) were generated using MZmine (v3.5.0). Molecular networking was performed using the GNPS Molecular Networking platform (https://gnps.ucsd.edu/ProteoSAFe/static/gnps-splash.jsp), with classical-based networking constructed from MS/MS fragmentation data. The default parameters were used, and the molecular network was visualized on the Cytoscape software (v3.10.2).

### Isolation of Cyclopeptides

The flatline (TJLE 34.1) strain was point-inoculated on solid rice media for 2 weeks. The culture was lyophilized and extracted with 1.5 L methanol (MeOH) thrice to be removed by evaporation in vacuo. The crude extract was solvent–solvent partitioned with EtOAc and H_2_O at a 1:1 ratio (Figure S23). After forming a separate layer, the EtOAc layer was removed, and the H_2_O layer was collected and dried in vacuo to yield a residue. The H_2_O extract (∼10 g) was further separated using the CombiFlash MPLC on a disposable 220 g silica column with a chloroform (CHCl_3_)/CH_3_OH linear gradient method: 40–100% CH_3_OH to yield eight fractions. Fractions 5–7 were further purified a few times using a prep C-18 column on HPLC with a linear gradient separation method: 5–35% MeCN 0.1% formic acid to afford white amorphous 7.0 mg of **1** and 3.3 mg of MBJ-0110. The structures of **1** and MBJ-0110 were drawn by using ChemDraw 22.0.0. The -dimensional (3D) model of **1** was generated through a geometric optimization using the PM3 semiempirical method in Spartan 10 and visualized in PyMOL 3.1.4.1. In the 3D image, key ROESY correlations were depicted, highlighting H-2, H-8, and the chair conformation of one of the piperidine rings (eq = equatorial, ax = axial).

### 3-Hydroxy-MBJ-0110 (1)

A colorless amorphous powder; [α]_D_^24^ −26 (*c* 0.10, MeOH); UV (MeOH) end; IR (ν_max_) 1500, 1610 cm^–1^; ^1^H and ^13^C NMR data, Table 2; HR-ESI-MS *m*/*z* 580.2964 [M + H]^+^ (calcd for C_27_H_42_N_5_O_9_, 580.2983).

### Advanced Marfey’s Analysis

Advanced Marfey’s analysis^32^ was conducted to determine the absolute configuration of compound **1**. About 0.2 mg of **1** was hydrolyzed with 1 mL of 6 M hydrochloric acid (HCl) at 110 °C for 4 h into a small glass vial. The resulting hydrolysate was dried under air flow, resuspended with 50 μL of H_2_O, and derivatized with 100 μL of 1% 1-fluoro-2,4-dinitrophenyl-5-leucine-amide (d,l-FDLA and l-FDLA) in acetone. Then, 20 μL of 1 M sodium bicarbonate (NaHCO_3_) was added, and the mixture was agitated at 40 °C for 1 h. The derivatized mixture was neutralized with 10 μL of 2 M HCl, diluted with 200 μL MeOH, and transferred to an LC–MS vial. Amino-acid standards l-Pip, l-Pro, and l-Asp (0.2–1 mg) were derivatized with d,l-FDLA and l-FDLA in a similar manner. The Marfey’s derivatives were analyzed using a UHPLC–MS system with 5 μL injection. The analytical method was developed with a linear gradient system of H_2_O/MeCN with 0.05% formic acid (10–60% MeCN, 40 min; flow rate, 0.2 mL/min). The retention times of FDLA-derivatized hydrolysate **1** were compared with the retention times of the derivatized amino acid standards.

### Genome Mining and Synteny Analysis of the mbj BGC

We ran the Web server version of antiSMASH (v8.0.2)^57^ to generate the initial list of 24 NRPS containing BGCs in our *P. expansum* D1 isolate. The list of 24 candidate NRPSs was narrowed down to two by looking for NRPSs with exactly five modules (A-T-C domains = 1 module). To determine which other species contain syntenic genomic loci to that of the *mbj* BGC, we used a mix of local versions cBlaster (v1.3.18)^41^ and the online Web server CageCat (CompArative GEne Cluster Analysis Toolbox).^58^ The local database was run complementary to CageCat to ensure species with all or part of the mbj cluster were not missed due to NCBI api query limits that can sometimes limit the number of genomes analyzed when using CageCat. The local database was made as follows: 371 primarily Ascomycota genomes were downloaded using the NCBI data sets. We took care to select a diversity of species spanning the major Ascomycota taxonomic classes and included a small number of Basidiomycetes in addition to a single Mucoromycete and Chytridiomycota genome. Visualizations and analysis of the protein domains within each individual NRPS were performed using synthaser (v1.1.22)^42^ and PARAS (v1.0.0).^40^

### Generating Sequence Similarity Alignments

Selected homologous protein sequences to MbjA were exported to the FASTA format using the Biopython (v1.85) library.^59^ Pairwise global alignments were computed between the reference MbjA protein from *P. expansum* D1 and all identified homologues using the pairwise2.align.globalxx command (match score = 1, mismatch/gap penalties = 0). Sequence similarity was calculated as the alignment score normalized by the length of the shorter sequence in each pair to account for potential variation in protein length. Homologues were ordered by descending similarity to the reference, and the resulting similarity values were visualized as a heatmap using the Seaborn (v0.13.2)^60^ and matplotlib (v3.9.4)^61^ Python libraries.

### Reconstructing the Species Tree from the Locally Downloaded Genomes

We reconstructed a kingdom-level species tree by running a coalescent model, ASTRAL v5.7.8, on 290 BUSCO gene trees.^62,63^ Each gene tree was made as following: sequence alignment was performed with MAFFT v7.475 ^64^ with the “-auto” parameter. The alignments underwent trimming using trimAl v1.2,^65^ with the “-gappyout” parameter. Lastly, we made phylogenomic trees from the trimmed alignments using IQTree v2.0.3 ^66^ and ran 1000 ultrafast bootstrap replicates. We utilized ModelFinder^67^ to select the most suitable model of sequence evolution for each gene tree. The tree was rooted at *Spizellomyces punctatus*, which was the only Chytridiomycota genome included in our data set. All tree visualizations were created with the help of the ggtree (v3.11.0)^68^ and ggtreeExtra (v1.12.0)^69^ packages.

## Supplementary Material





## Data Availability

The NMR data for compound **1** have been deposited in the Natural Products Magnetic Resonance Database (NP-MRD; www.np-mrd.org) and can be found at NP0352144.

## Notes

General information on strains, OSMAC analysis, HR–ESI–MS and HR–ESI–MS/MS data, 1D and 2D NMR spectra, IR spectra, LC–MS results from the Marfey’s analysis, Southern blot, and PCR confirmations (PDF)
Results from AntiSMASH of predicted BGCs of *P. expansum* Pe-21 (XLSX)
