# Supplementary material for: Genetic Dereplication of Multiple Penicillium expansum Biosynthetic Gene Clusters Reveals Cryptic Penta-Cyclopeptide Production
Source: J Nat Prod. 2026 Feb 24;89(3):912–24. doi: 10.1021/acs.jnatprod.5c01531 (PMC13036780; doi:10.1021/acs.jnatprod.5c01531)
Supplement: Supplementary file 1 [file np5c01531_si_001.pdf]

Supplementary data 1

Genetic dereplication of multiple *Penicillium expansum* biosynthetic gene clusters reveals cryptic penta-cyclopeptide production

Mira Syahfriena Amir Rawa[1], Benjamin J. Haefner[2], Grant R. Nickles[1], Enrique Aguilar-Ramírez[1], Nancy P. Keller[1,2,*], and Justin L. Eagan[1,*]

[1]Department of Medical Microbiology and Immunology, University of Wisconsin-Madison, Madison, WI 53706, USA
[2]Department of Plant Pathology, University of Wisconsin-Madison, Madison, WI 53706, USA

[*]Corresponding authors
Email: jeagan@wisc.edu
Email: npkeller@wisc.edu

KEYWORDS: *Penicillium expansum*, genetic dereplication, cyclopeptide, non-ribosomal peptide synthase

Contents:

Table S1. List of *P. expansum* strains developed in this study./ page 3
Figure S1. Citrinin production is not completely abolished by deleting the in-cluster transcription factor, *ctnA*. / page 4
Figure S2. Total ion chromatograms (TIC) of *P. expansum* WT (TJT14.1) and flatline (TJLE 34.1) strains on PDA and extracted ion chromatograms (EIC) of citrinin, roquefortine C, communesin B, andrastin B, and patulin peaks. / page 5
Table S2. Chemical information of SMs of *P. expansum* deleted in this study. / page 6
Table S3. Media used in this study. / page 7
Figure S3. Phenotype of WT in comparison to the flatline strain grown in 15 different culture conditions. / page 8
Figure S4. TICs (+ ion mode) of WT (left) vs. flatline (right) strains cultivated in 15 different culture conditions. / page 9–10
Figure S5. EICs (+ ion mode) of compounds **1** (*m/z* 580), **2** (*m/z* 550), and **3** (*m/z* 564) from *P. expansum* WT and flatline strains grown on PDA or rice. / page 11–12
Figure S6. FT-IR spectrum of compound **1** (MeOH). / page 13
Figure S7. HR-ESI-MS and HR-ESI-MS/MS spectra of compound **1**. / page 14
Figure S8. HR-ESI-MS and HR-ESI-MS/MS spectra of MBJ-0110 (**2**). / page 15
Figure S9. ^{1}H NMR spectrum of **3**. / page 16
Figure S10. ^{13}C NMR spectrum of **3**. / page 17
Figure S11. HSQC NMR spectrum of **3**. / page 18
Table S4. NMR data comparison of **3** from this study and previous report./ page 19
Figure S12. ^{1}H NMR spectrum of **1**. / page 20
Figure S13. ^{13}C NMR spectrum of **1**. / page 21
Figure S14. DEPT-135 NMR spectrum of **1**. / page 22
Figure S15. HSQC NMR spectrum of **1**. / page 23
Figure S16. COSY NMR spectrum of **1**. / page 24
Figure S17. HMBC NMR spectrum of **1**. / page 25
Figure S18. Spin system of hydroxy-Ile of **1** based on HMBC NMR correlations. / page 26
Figure S19. Total ion chromatograms of advanced Marfey's analysis of **1** compared to amino acid standards derivatized with *L*-FDLA or *D,L*-FLDA. / page 27
Table S5. Retention times of FDLA derivatives of amino acids derived from **1** and standards. / page 28
Figure S20. ROESY NMR spectrum and spin system of hydroxy-Ile of **1**. / page 29
Figure S21. HR-ESI-MS and HR-ESI-MS/MS spectra of **2**. / page 30
Figure S22. Southern blot and PCR confirmations of all deletion strains in this study. / page 31–34
Table S6. Primer sequences designed in this study. / page 35
Figure S23. Isolation chart of cyclopeptide **1** and MBJ-0110. / page 36

Table S1. List of *P. expansum* strains developed in this study.

Strain	Genotype	Parent
**TJT 14.1**	Δ*ku70*::six-site	TDL 2.4
**TJLE 1.1**	Δ*ku70*::six-site, Δ*ctnA*::*βrec/hph*	TJT 14.1
**MJLE 1.1/2/3**	Δ*ku70*::six-site, Δ*ctnA*::six-site	TJLE 1.1
**TJLE 4.1/2**	Δ*ku70*::six-site, Δ*ctnA*::six-site, Δ*patL*::*βrec/hph*	MJLE 1.1
**MJLE 2.1/2**	Δ*ku70*::six-site, Δ*ctnA*::six-site, Δ*patL*::six-site	TJLE 4.1
**TJLE 16.1/2/3**	Δ*ku70*::six-site, Δ*ctnA*::six-site, Δ*patL*::six-site, Δ*citS*::*βrec/hph*	MJLE 2.1
**MJLE 3.1/2/3**	Δ*ku70*::six-site, Δ*ctnA*::six-site, Δ*patL*::six-site, Δ*citS*:six-site	TJLE 16.1
**TJLE 17.1/2/3**	Δ*ku70*::six-site, Δ*ctnA*::six-site, Δ*patL*::six-site, Δ*citS*:six-site, Δ*roqA*::*βrec/hph*	MJLE 3.1
**MJLE 4.1/2/3**	Δ*ku70*::six-site, Δ*ctnA*::six-site, Δ*patL*::six-site, Δ*citS*:six-site, Δ*roqA*::six-site	TJLE 17.1
**TJLE 27.1/2/3**	Δ*ku70*::six-site, Δ*ctnA*::six-site, Δ*patL*::six-site, Δ*citS*:six-site, Δ*roqA*::six-site, Δ*cns*::*βrec/hph*	MJLE 4.1
**MJLE 5.1/2/3**	Δ*ku70*::six-site, Δ*ctnA*::six-site, Δ*patL*::six-site, Δ*citS*:six-site, Δ*roqA*::six-site, Δ*cns*::six-site	TJLE 27.1
**TJLE 30.1/2/3**	Δ*ku70*::six-site, Δ*ctnA*::six-site, Δ*patL*::six-site, Δ*citS*:six-site, Δ*roqA*::six-site, Δ*cns*BGC::six-site, ΔPEX2_030390::*βrec/hph*	MJLE 5.1
**MJLE 6.1/2/3**	Δ*ku70*::six-site, Δ*ctnA*::six-site, Δ*patL*::six-site, Δ*citS*:six-site, Δ*roqA*::six-site, Δ*cns*BGC::six-site, ΔPEX2_030390::six-site	TJLE 30.1
**TJLE 34.1/2/3 (flatline)**	Δ*ku70*::six-site, Δ*ctnA*::six-site, Δ*patL*::six-site, Δ*citS*:six-site, Δ*roqA*::six-site, Δ*cns*BGC::six-site, ΔPEX2_030390::six-site, Δ*adrD*::*βrec/hph*	MJLE 6.1
**TBJH2.1/2/3**	Δ*ku70*::six-site, Δ*ctnA*::six-site, Δ*patL*::six-site, Δ*citS*:six-site, Δ*roqA*::six-site, Δ*cns*BGC::six-site, ΔPEX2_030390::six-site, Δ*PEXP_085540*::*βrec/hph*	MJLE 6.1
**TBJH3.1/2/3**	Δ*ku70*::six-site, Δ*ctnA*::six-site, Δ*patL*::six-site, Δ*citS*:six-site, Δ*roqA*::six-site, Δ*cns*BGC::six-site, ΔPEX2_030390::six-site, Δ*mbjA*::*βrec/hph*	MJLE 6.1

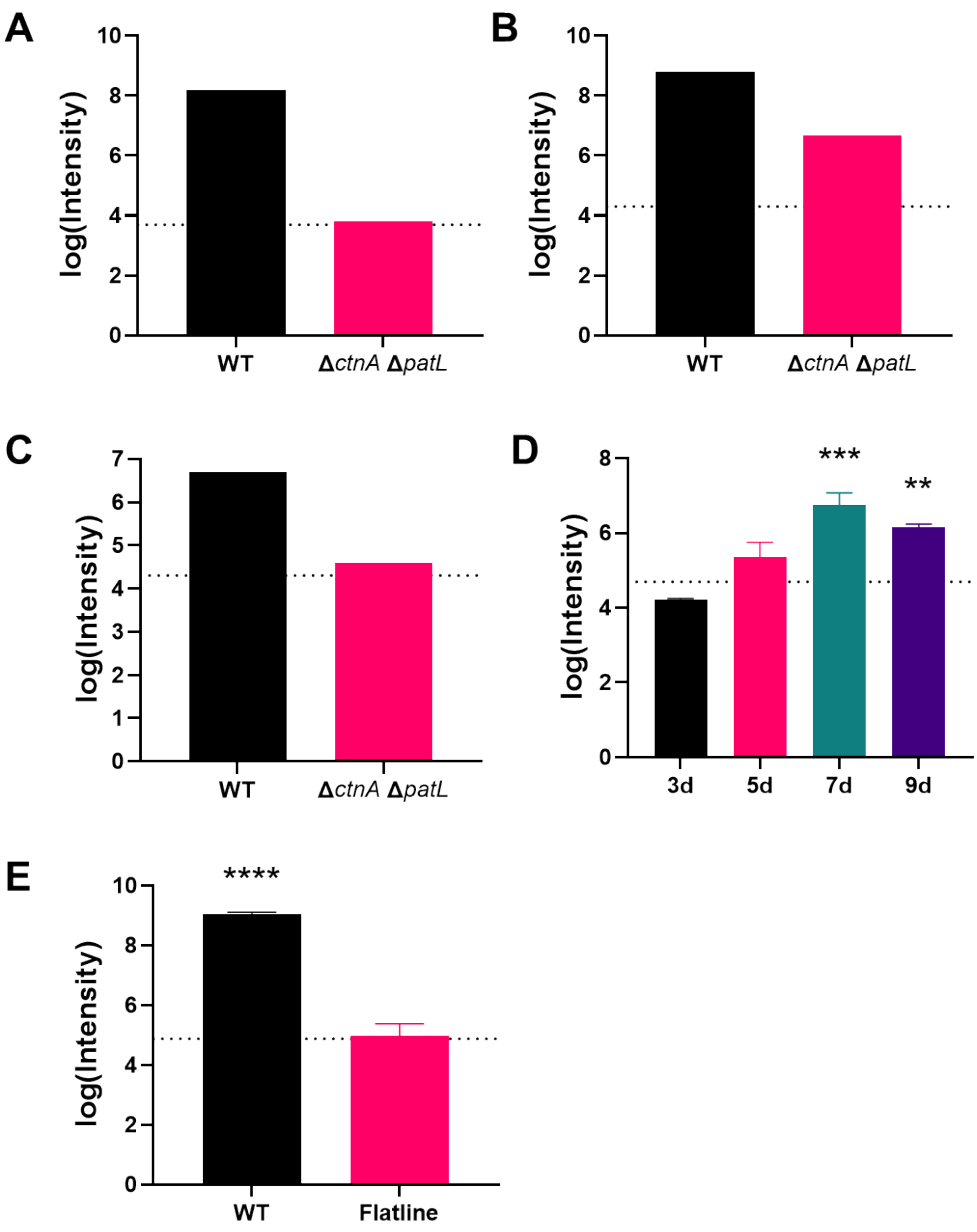

Figure S1. Citrinin production is not completely abolished by deleting the in-cluster transcription factor, *ctnA*. Analyses for figures S1A-B and S1D-E are liquid chromatography coupled with tandem mass spectrometry (LC-MS/MS) spectra filtered to *m/z* 251.0914 for citrinin signal intensity using Maven (version 2.0.1). Analysis for figure S1C is LC-MS/MS spectra filtered to *m/z* 155.0338 using Maven (version 2.0.1). The dashed line in all figures represents background signals at this *m/z* from a methanol control sample. Ordinary one-way ANOVA analyses by Prism's GraphPad (version 10.2.3) were performed for figures S1C and S1D**. A)** Pooled 200 potato dextrose agar (PDA) plates of wild type (WT) and ΔctnA ΔpatL (TJLE4) after 14 days of culture. **B)** Pooled 20 PDA plates of WT and TJLE4 after 10 days of culture. **C)** Pooled 20 PDA plates of WT and TJLE4 after 10 days of culture. **D)** Time course experiment of WT in potato dextrose broth (PDB) in triplicate after 3 days (3d), 5 days (5d), 7 days (7d), and 9 days (9d). 7d and 9d samples are significantly different from the baseline methanol control value ($p<0.001$ and $p<0.01$, respectively). **E)** WT and flatline (TJLE34) strains cultured on PDA for 7 days in triplicate.

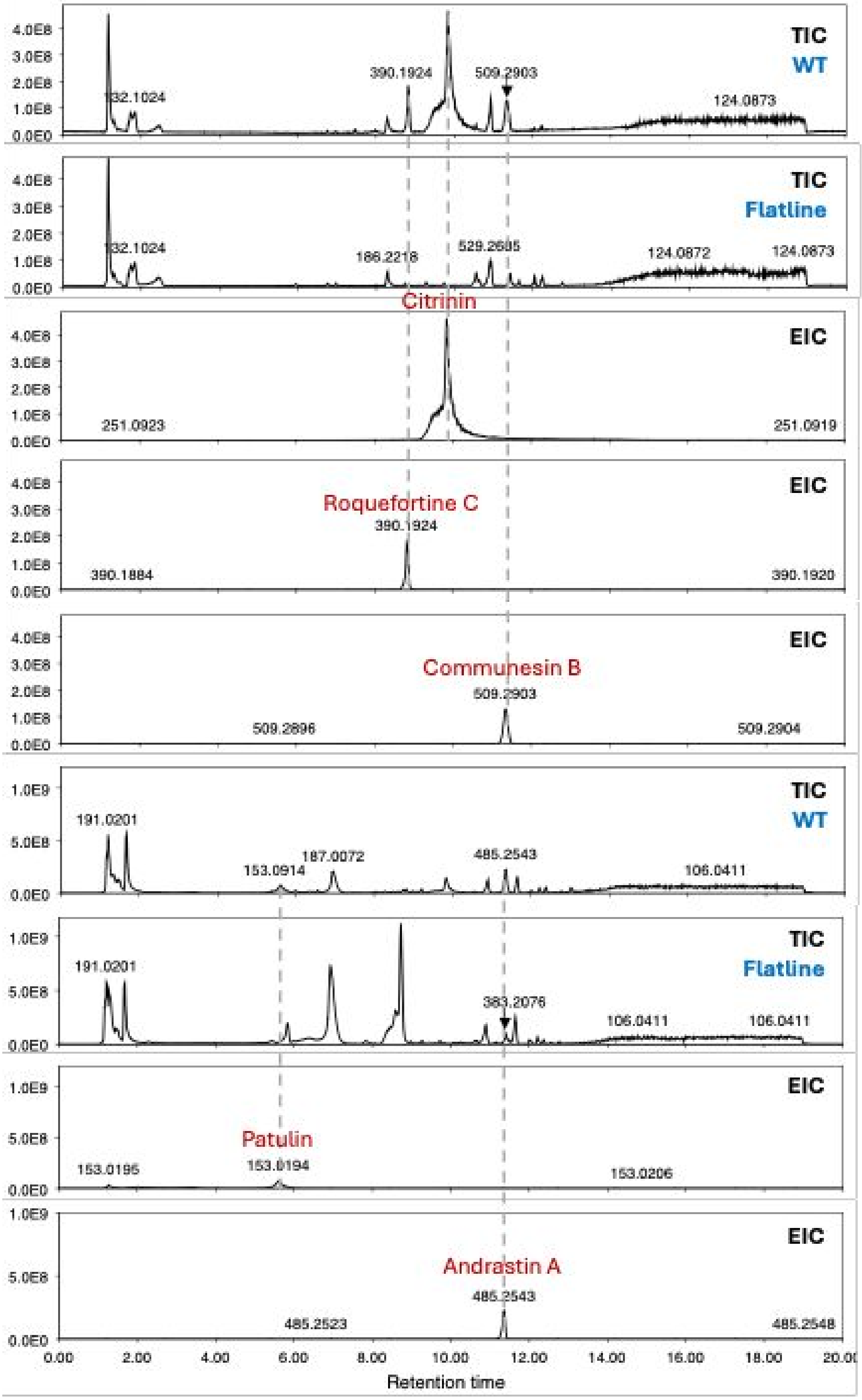

Figure S2. Total ion chromatograms (TIC) of *P. expansum* WT (TJT14.1) and flatline (TJLE 34.1) strains on PDA and extracted ion chromatograms (EIC): in + ion mode (citrinin, roquefortine C, and communesin B); in – ion mode (patulin, and andrastin A). These five target peaks were not observed in the flatline strain.

Table S2. Chemical information of SMs of *P. expansum* deleted in this study.

No.	SMs	Class	Molecular Formula	*m/z* values	r.t. (min)	Chemical Structure
**1**	Patulin	Polyketide	$C_7H_6O_4$	153.0199 $(M\text{-}H)^-$	5.61 min	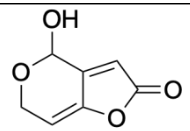
**2**	Citrinin	Polyketide	$C_{13}H_{14}O_5$	251.0914 $(M+H)^+$	9.83 min	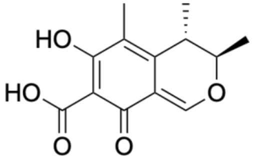
**3**	Roquefortines	Nonribosomal peptide (indole alkaloid)	(C) $C_{22}H_{23}N_5O_2$<br>(D) $C_{22}H_{25}N_5O_2$	390.1925 $(M+H)^+$<br>392.2096 $(M+H)^+$	8.81 min<br>7.97 min	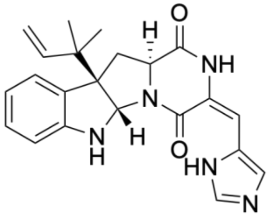<br>Roquefortine C
**4**	Communesins	Nonribosomal peptide (indole alkaloid)	(A) $C_{28}H_{32}N_4O_2$<br>(B) $C_{32}H_{36}N_4O_2$<br>(E/F) $C_{28}H_{32}N_4O$	457.2598($M+H)^+$<br>509.2911 $(M+H)^+$<br>441.2648 $(M+H)^+$	9.77 min<br>11.33 min<br>9.17 min	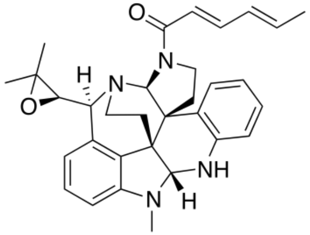<br>Communesin B
**5**	Andrastins	Meroterpenoid (terpene + polyketide)	(A) $C_{28}H_{38}O_7$<br>(B) $C_{28}H_{40}O_7$<br>(C) $C_{28}H_{40}O_6$<br>(D) $C_{26}H_{36}O_5$<br>(E/F) $C_{26}H_{36}O_5$	487.2686 $(M+H)^+$ / 485.2542 $(M\text{-}H)^-$<br>489.2838 $(M+H)^+$ / 487.2700 $(M\text{-}H)^-$<br>473.2890 $(M+H)^+$ / 471.2752 $(M\text{-}H)^-$<br>429.2653 $(M+H)^+$ / 427.2490 $(M\text{-}H)^-$<br>453.2622 $(M+Na)^+$	11.37 min<br>10.57 min<br>13.04 min<br>11.93 min<br>11.27/12.46 min	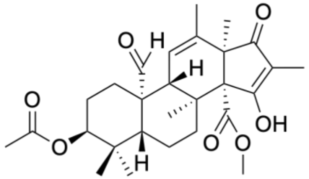<br>Andrastin A

Table S3. Media used in this study.

Name	Acronym	Reference
Glucose minimal media	GMM	[1]
Potato dextrose agar	PDA	Purchased from Sigma-Aldrich, MI, USA
Czapek yeast extract agar	CYA	[2]
Glucose yeast extract trace elements	CHAMPS	[3]
Malt extract agar	MEA	Purchased from Millapore Sigma, MA, USA
Yeast extract sucrose	YES	[4]
Yeast extract peptone	YEP	[4]
Casamino acids glucose peptone yeast extract	CPGY	[5]
Oats	N/A	5 g of oats in 7 mL of ddH_2O, autoclaved
Rice	N/A	5 g of rice in 7 mL of ddH_2O, autoclaved

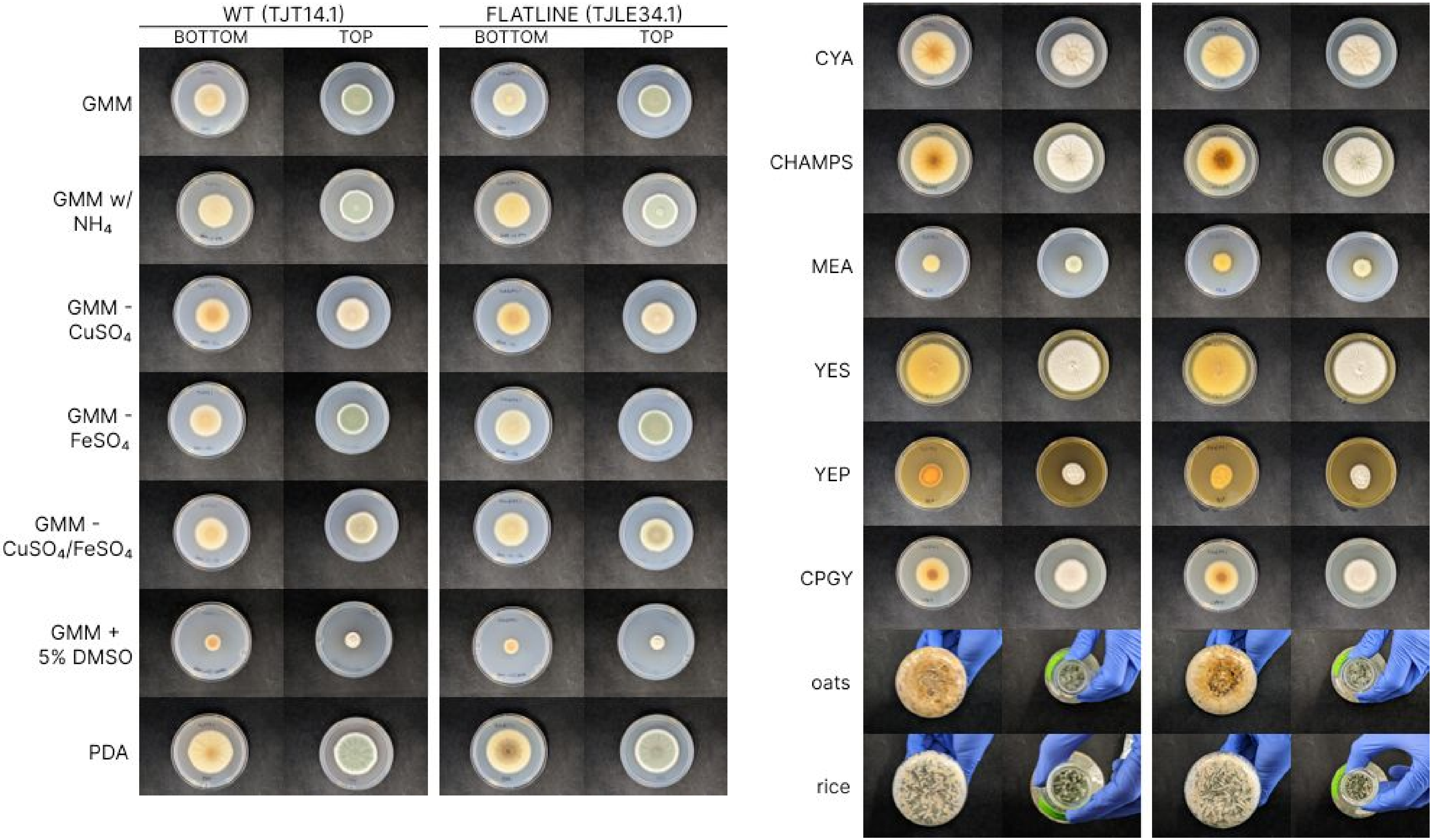

Figure S3. Phenotype of wild-type (WT) in comparison to the flatline strain grown in 15 different culture conditions.

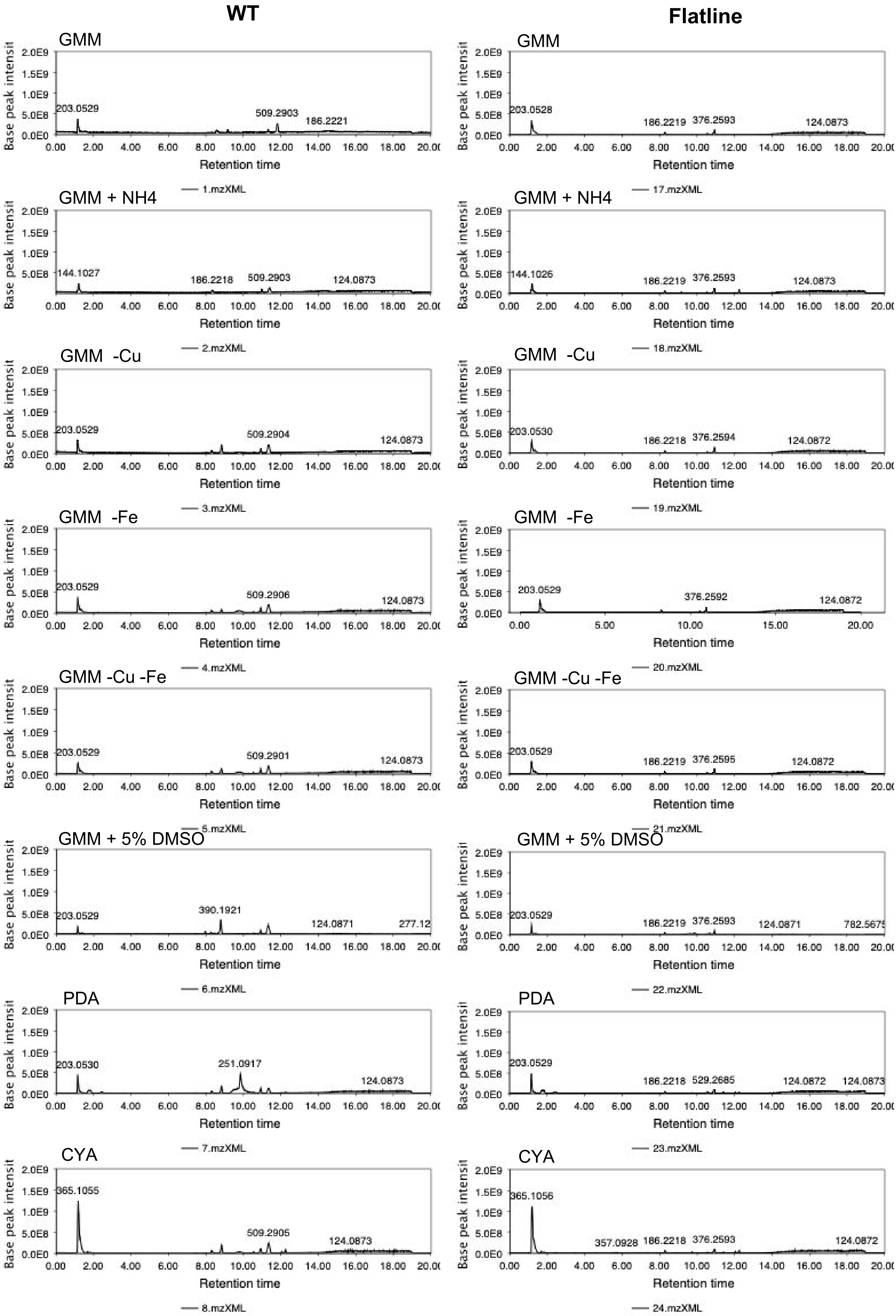
WT
Flatline
GMM
GMM + NH4
GMM -Cu
GMM -Fe
GMM -Cu -Fe
GMM + 5% DMSO
PDA
CYA
Base peak intensit
Retention time
203.0529
509.2903
186.2221
203.0528
186.2219
376.2593
124.0873
144.1027
186.2218
144.1026
509.2904
203.0530
376.2594
124.0872
509.2906
203.0529
376.2592
509.2901
376.2595
390.1921
124.0871
277.12
782.5675
251.0917
529.2685
365.1055
509.2905
365.1056
357.0928
1.mzXML
2.mzXML
3.mzXML
4.mzXML
5.mzXML
6.mzXML
7.mzXML
8.mzXML
17.mzXML
18.mzXML
19.mzXML
20.mzXML
21.mzXML
22.mzXML
23.mzXML
24.mzXML

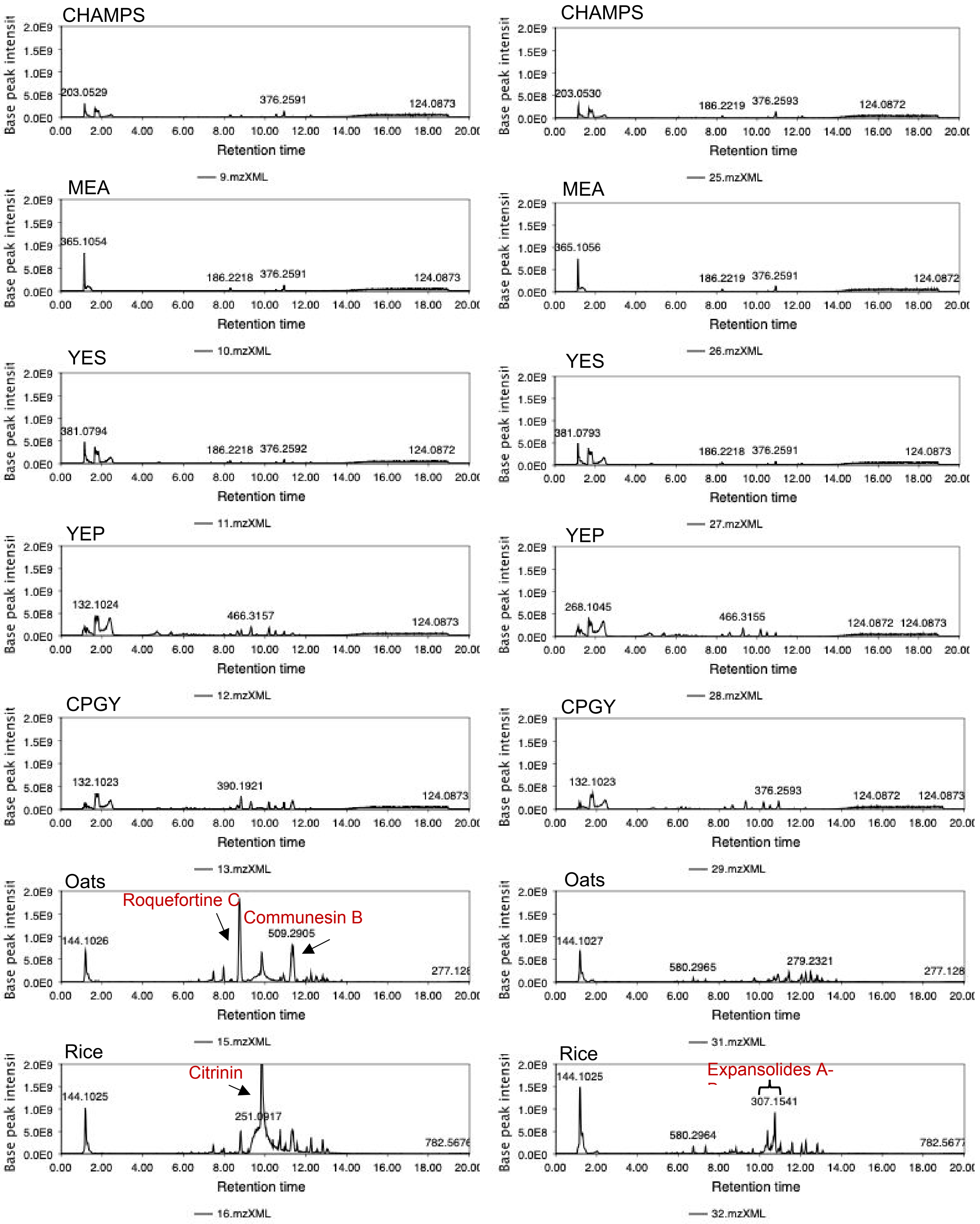

Figure S4. TICs (+ ion mode) of WT (left) vs. flatline (right) strains cultivated in 15 different culture conditions. Chemical diversity and productions were increased when grown on grain-based media like oats and rice. The maximum base peak intensity on y-axis was standardized to 2×10^9.

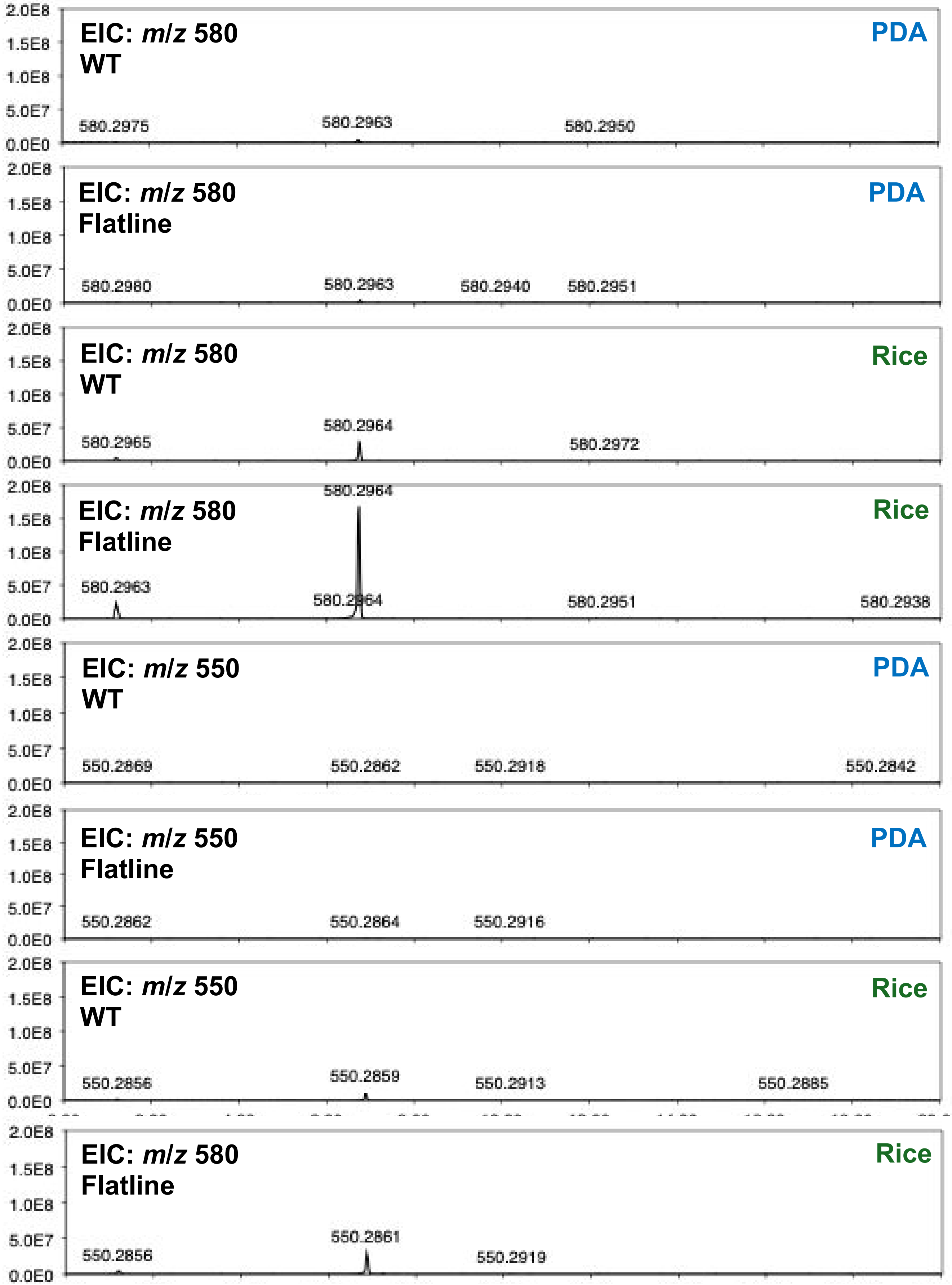
EIC: m/z 580
WT
PDA
580.2975
580.2963
580.2950
EIC: m/z 580
Flatline
PDA
580.2980
580.2963
580.2940
580.2951
EIC: m/z 580
WT
Rice
580.2965
580.2964
580.2972
EIC: m/z 580
Flatline
Rice
580.2964
580.2963
580.2964
580.2951
580.2938
EIC: m/z 550
WT
PDA
550.2869
550.2862
550.2918
550.2842
EIC: m/z 550
Flatline
PDA
550.2862
550.2864
550.2916
EIC: m/z 550
WT
Rice
550.2856
550.2859
550.2913
550.2885
EIC: m/z 580
Flatline
Rice
550.2856
550.2861
550.2919
2.0E8
1.5E8
1.0E8
5.0E7
0.0E0

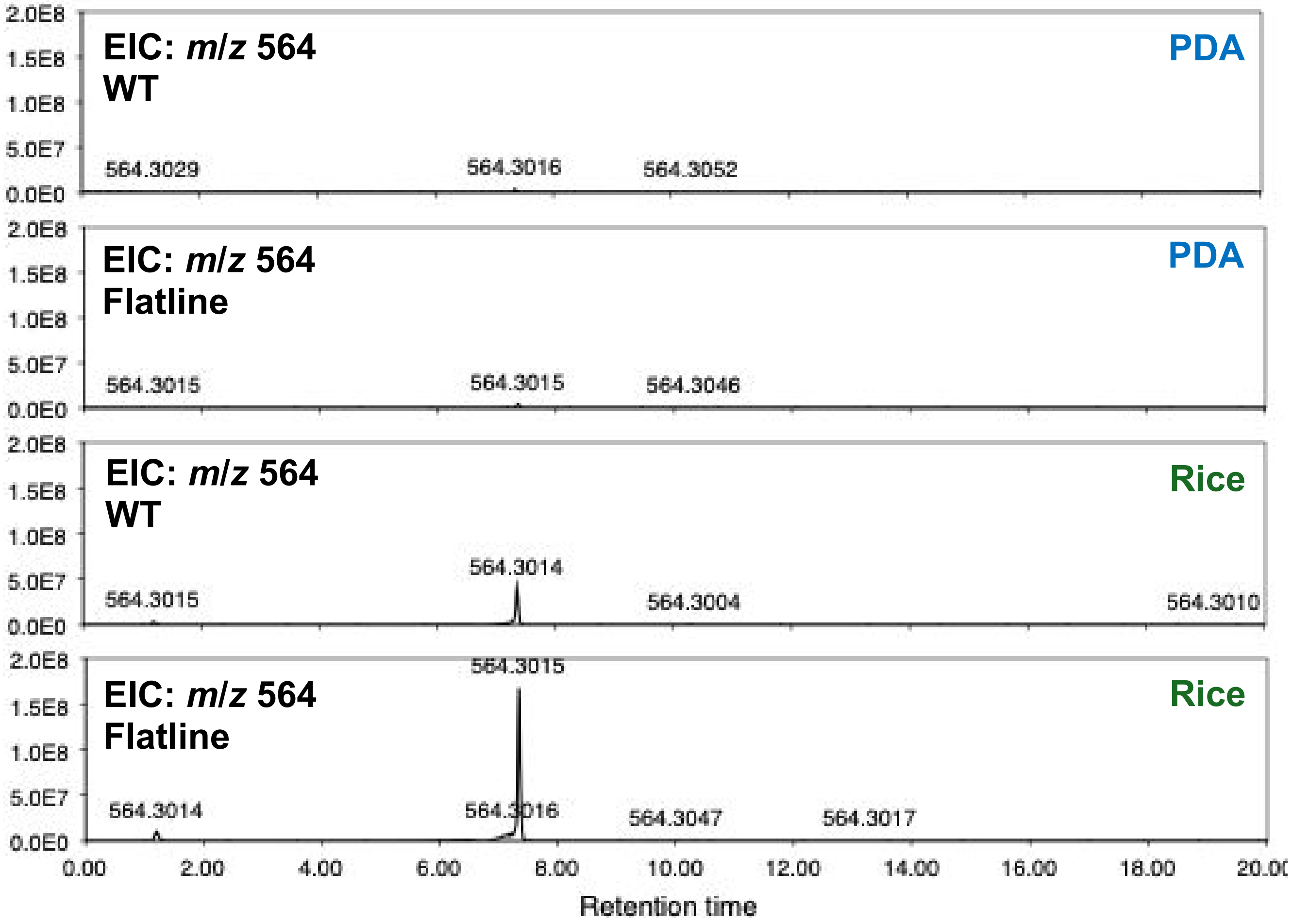

Figure S5. EICs (+ ion mode) of compounds **1** (*m*/*z* 580), **2** (*m*/*z* 550), and **3** (*m*/*z* 564) from *P. expansum* WT and flatline strains grown on PDA or rice. Productions of **1**–**3** were greater in flatline than in WT and when grown on rice. The maximum base peak intensity on y-axis was set to $2{\times}10^{8}$.

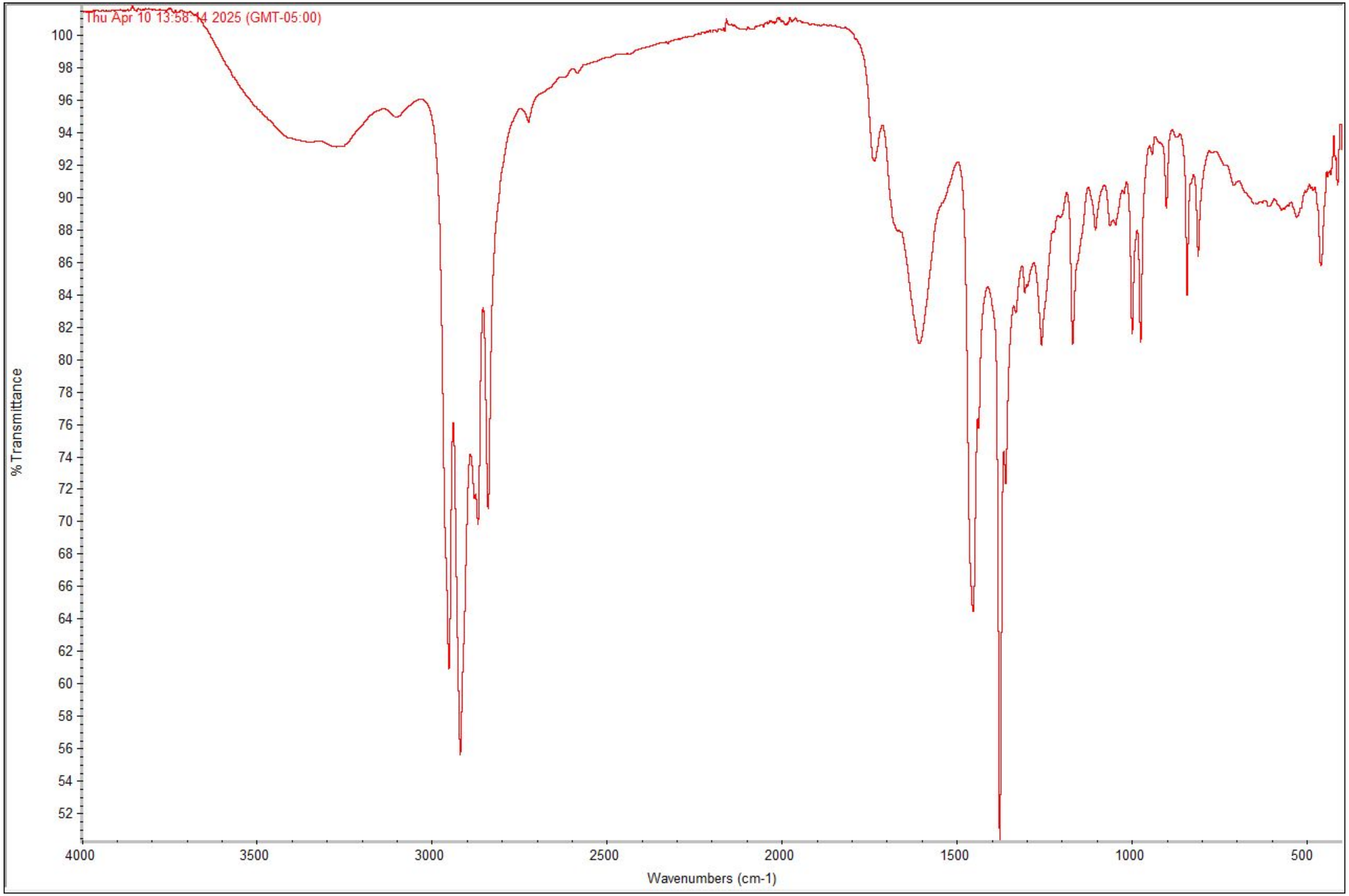

Figure S6. FT-IR spectrum of compound **1** (MeOH).

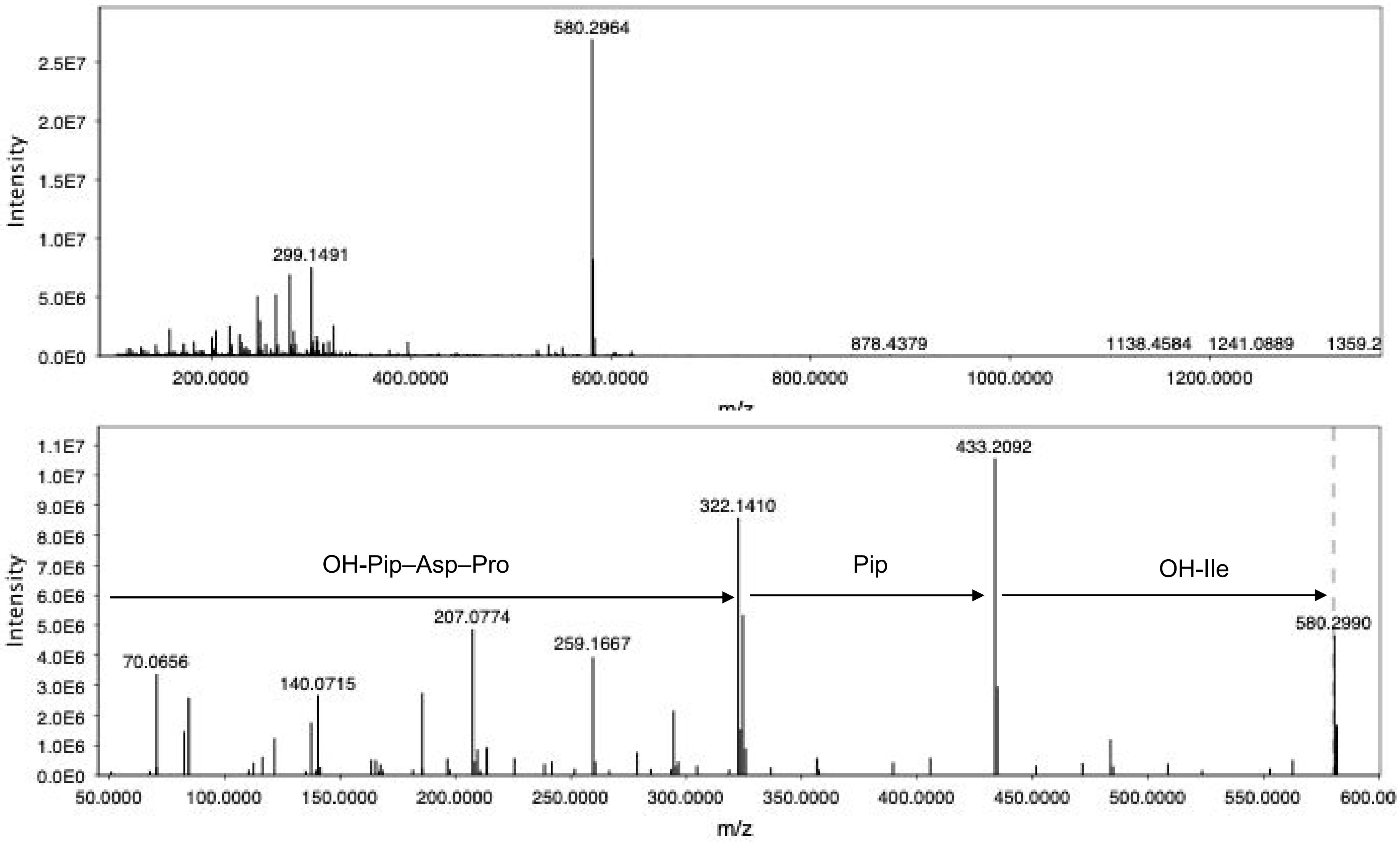

Figure S7. HR-ESI-MS and HR-ESI-MS/MS spectra of compound **1**.

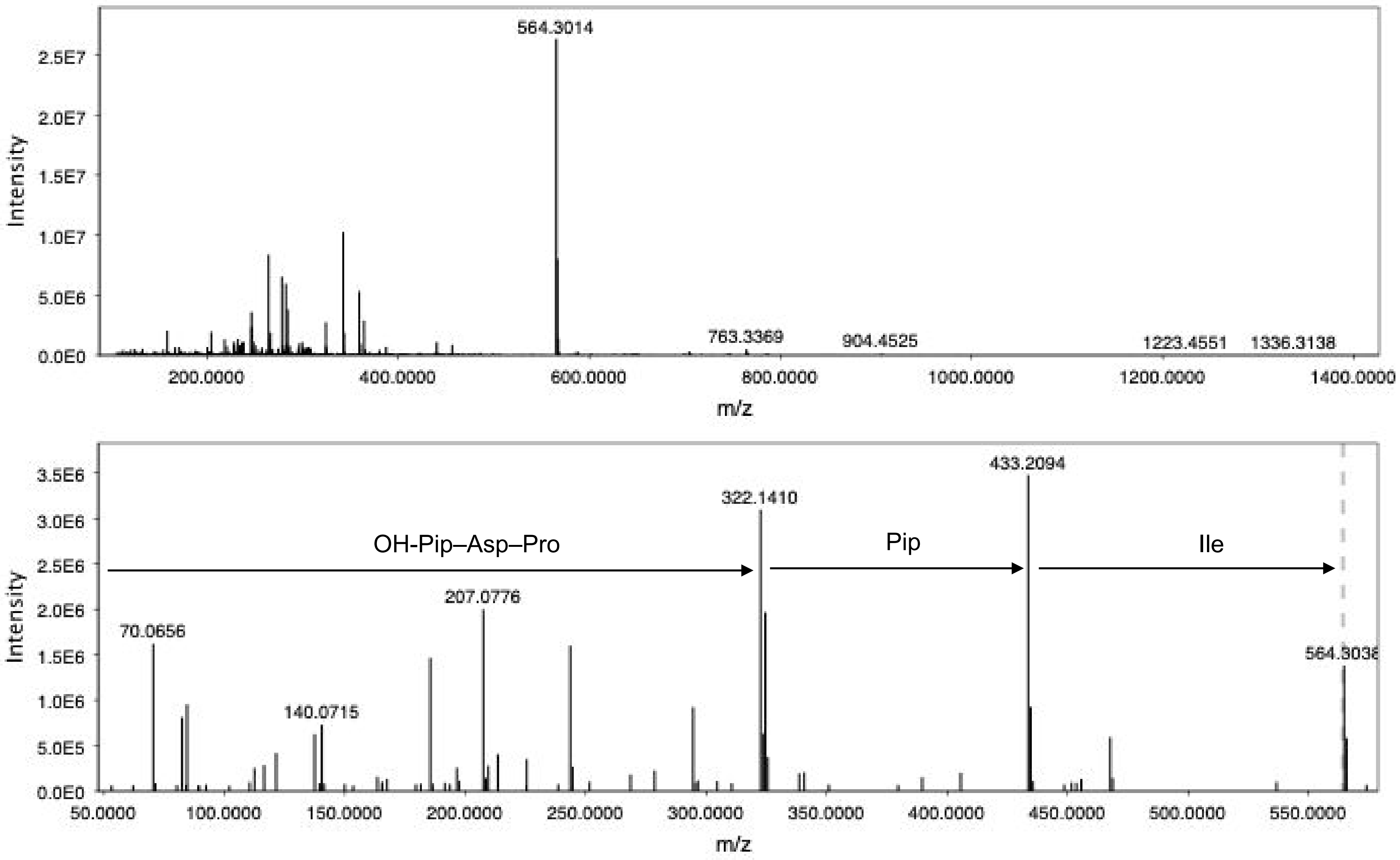

Figure S8. HR-ESI-MS and HR-ESI-MS/MS spectra of MBJ-0110 (**3**).

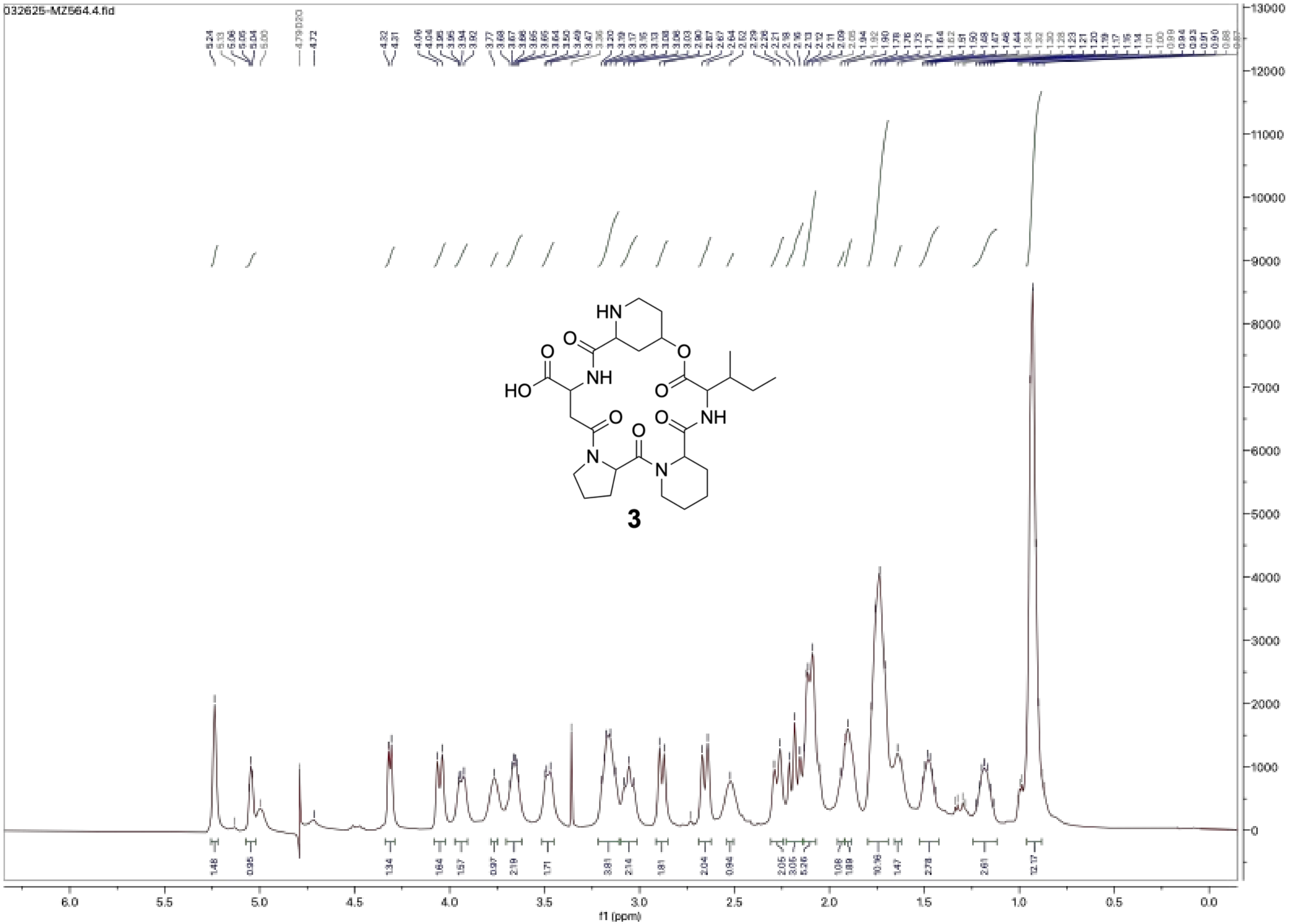

Figure S9. ^{1}H NMR spectrum of **3** in D_2O (500 MHz). The D_2O solvent peak was suppressed.

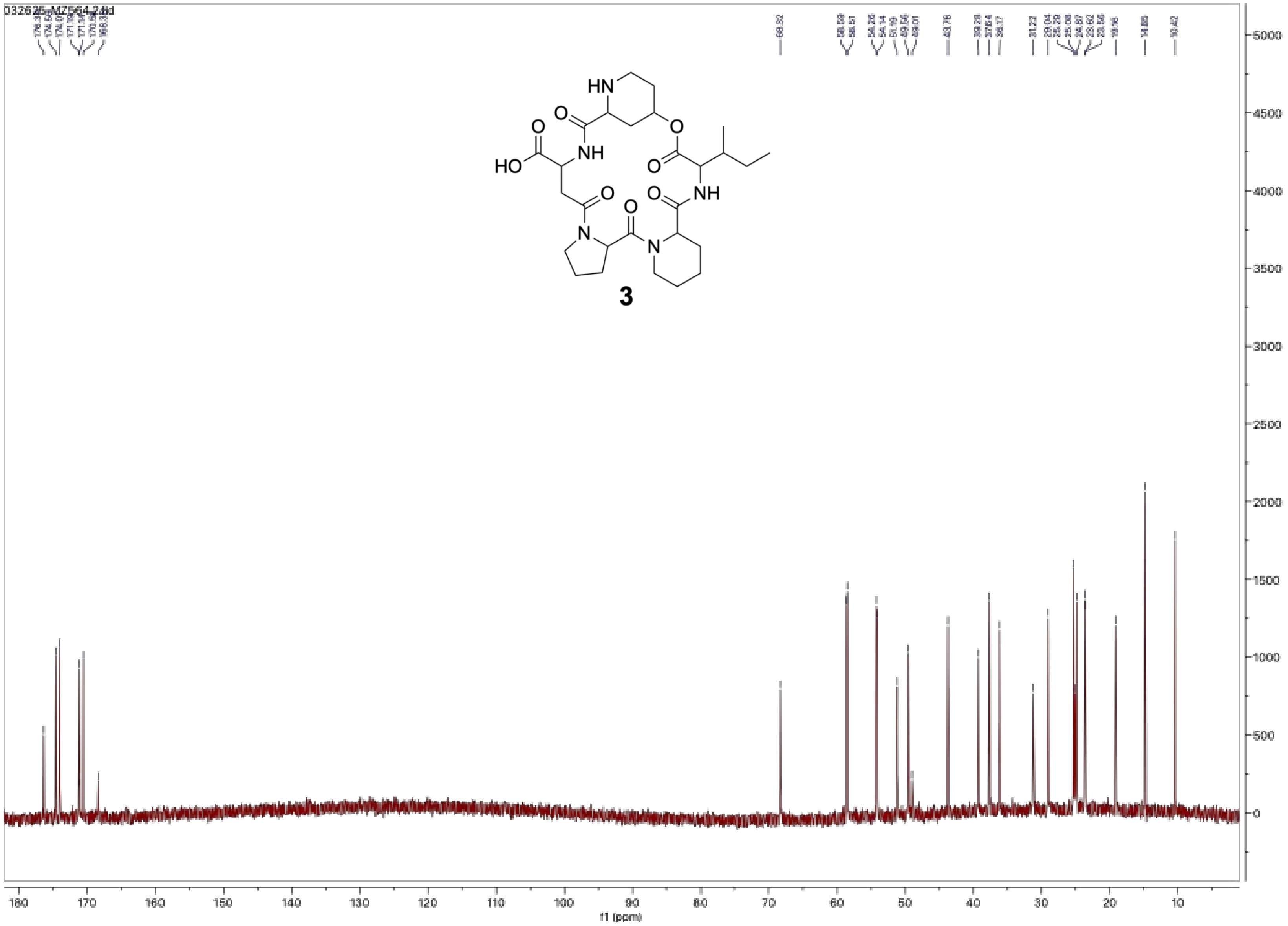

Figure S10. ^{13}C NMR spectrum of **3** in D_2O (125 MHz).

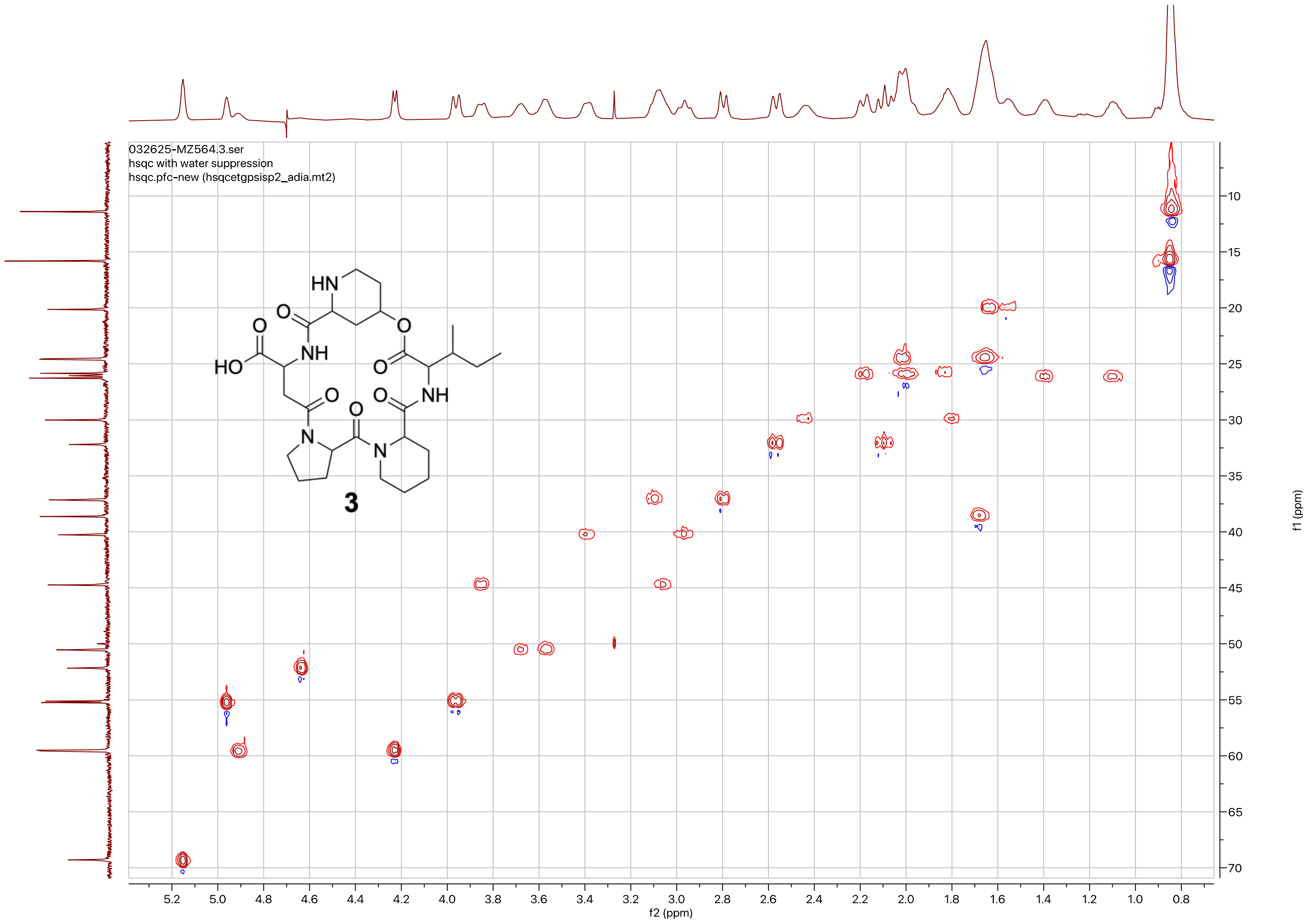

Figure S11. HSQC NMR spectrum of **3** in D_2O (^1H: 500 MHz, ^{13}C: 125 MHz).

Table S4. NMR data comparison of **3** from this study and previous report.

**Position**	**MBJ-0110 in CD_3OD (this study)**		**MBJ-0110 in CD_3OD (previous study[6])**	
	**δC**	**δH, mult. (*J* in Hz)**	**δC**	**δH, mult. (*J* in Hz)**
**1**	169.7	-	169.7	
**2**	59.2	4.36, d (7.5)	59.2	4.36, d (7.8)
**3**	40.2	1.68, overlap	39.7	1.68, overlap
**4**	26.7	1.51, m; 1.16 m	26.7	1.52, m; 1.19, m
**5**	11.5	0.94, t (7.6)	11.4	0.95, t (7.2)
**6**	15.9	0.93, d (6.9)	15.9	0.93, d (6.6)
**7**	173.1	-	173.0	
**8**	54.5	5.14, br. s	54.8	5.08, br s
**9**	25.0	2.19, m; 1.62, overlap	25.0	2.20, overlap; 1.62, overlap
**10**	21.0	1.78, overlap; 1.70, overlap	20.9	1.78, overlap; 1.70, overlap
**11**	25.5	1.73, overlap	25.5	1.73, overlap; 1.73, overlap
**12**	45.0	3.95, d (13.6); 2.97, overlap	45.0	3.97, br d (15.6); 3.00, overlap
**13**	175.7	-	175.9	
**14**	59.6	4.92, dd (4.2, 8.4)	59.7	4.95, dd (4.8, 8.4)
**15**	30.0	2.44, m; 1.79, m	30.0	2.47, m; 1.80, overlapl
**16**	26.3	2.05, m; 1.96, overlap	26.3	2.08, overlap; 1.96, overlap
**17**	50.2	3.71, overlap	50.2	3.76, overlap; 3.70, overlap
**18**	171.4	-	171.3	
**19**	37.4	3.06, m; 2.94, m	37.0	3.10, dd (7.2, 12.0); 2.96, overlap
**20**	50.2	4.74, m	51.7	4.82, m
**21**	175.7	-	175.7	
**22**	*	-	169.0	
**23**	55.9	3.89, d (12.4)	55.5	4.00, d (7.2)
**24**	30.0	2.53, d (14.1); 2.05, overlap	32.8	2.62, br d (13.8); 2.07, overlap
**25**	70.4	5.16, br. s	68.9	5.17, br s
**26**	26.7	2.19, m; 2.04, m	26.8	2.19, overlap; 2.02, overlap
**27**	41.0	3.18, m; 2.84, t (13.2)	40.6	3.39, br d (9.6); 2.98, overlap

*Signal not observed

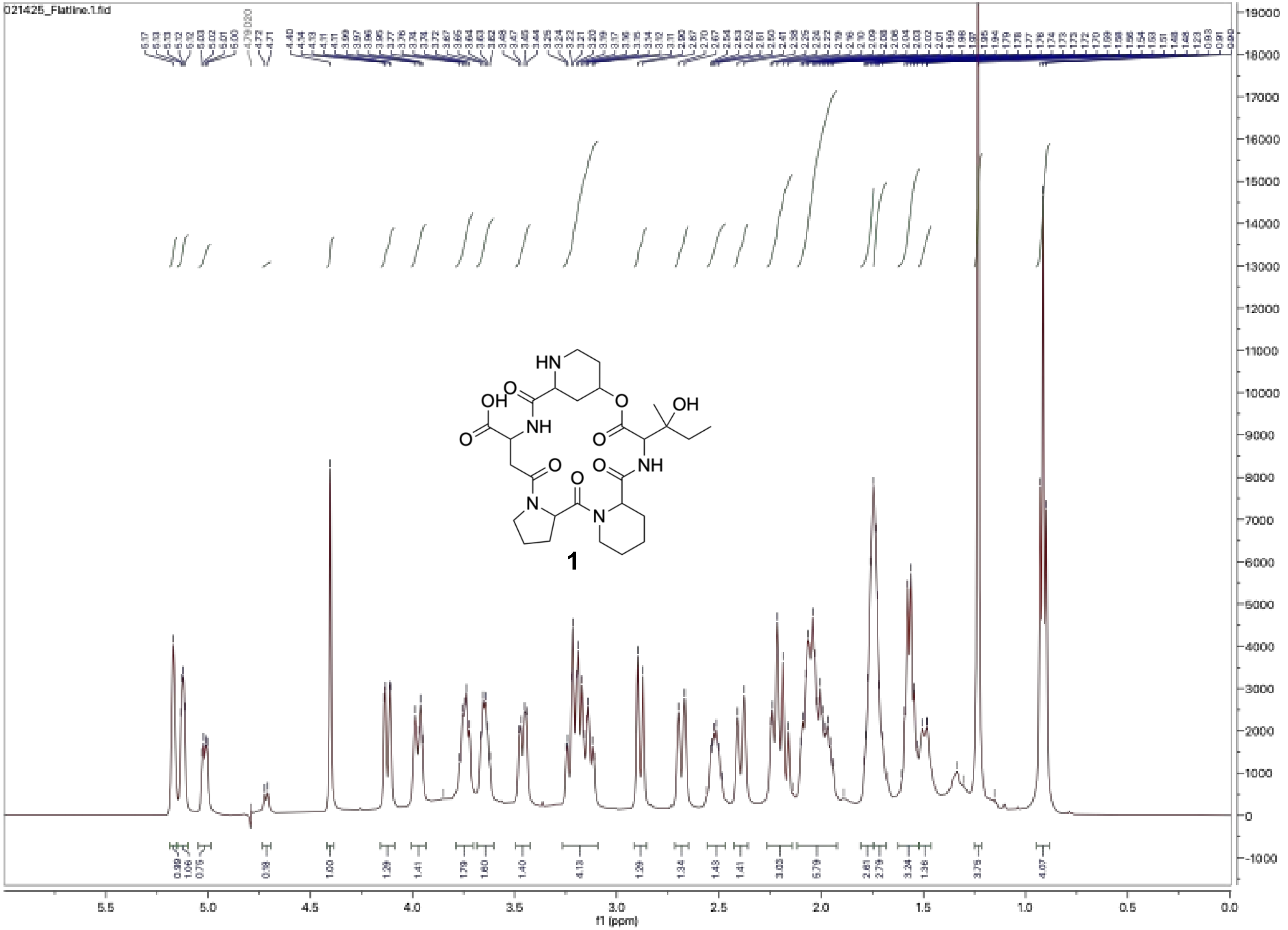

Figure S12. ^{1}H NMR spectrum of **1** in D_2O (500 MHz). The D_2O solvent peak was suppressed.

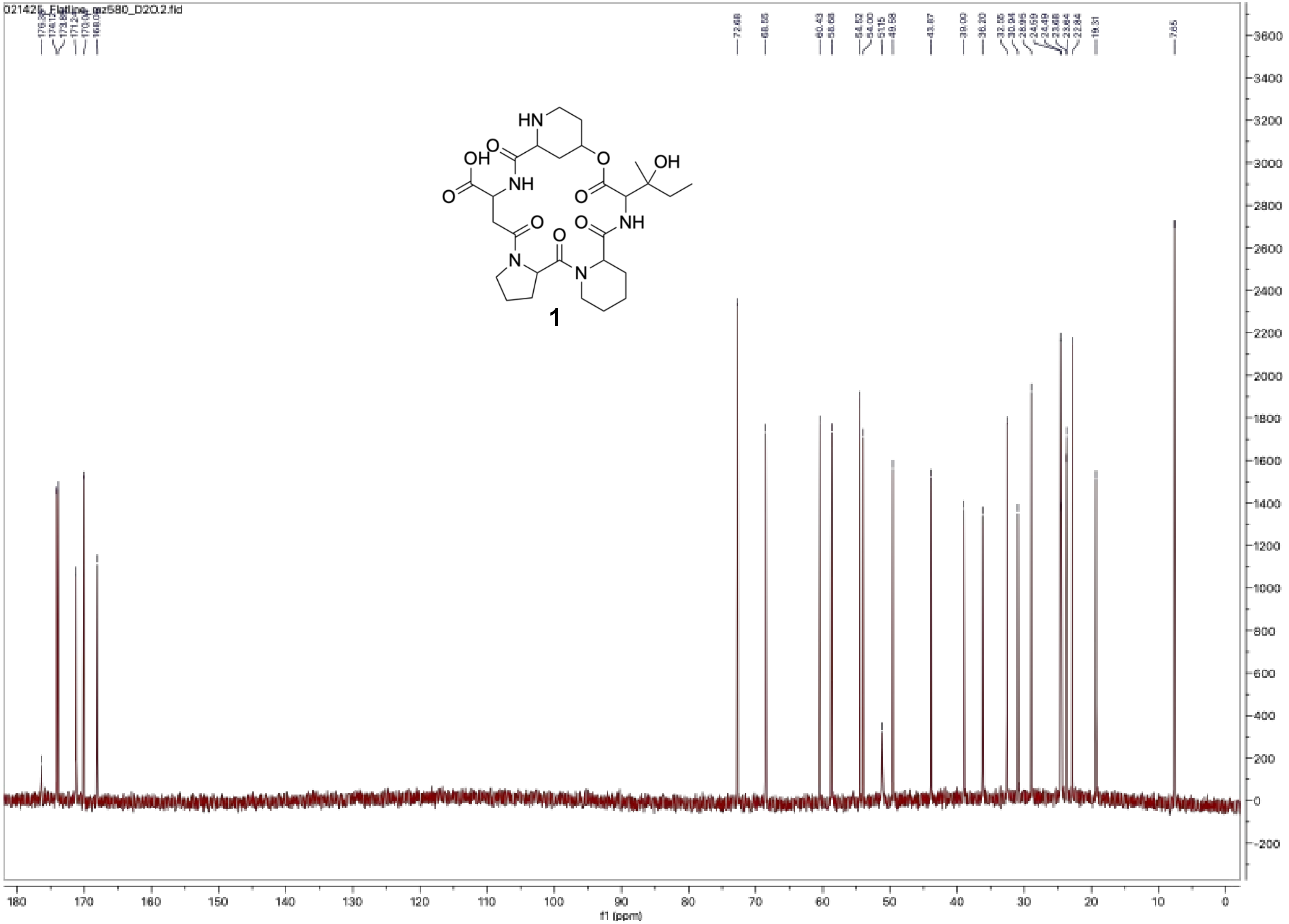

Figure S13. ^{13}C NMR spectrum of **1** in D_2O (125 MHz).

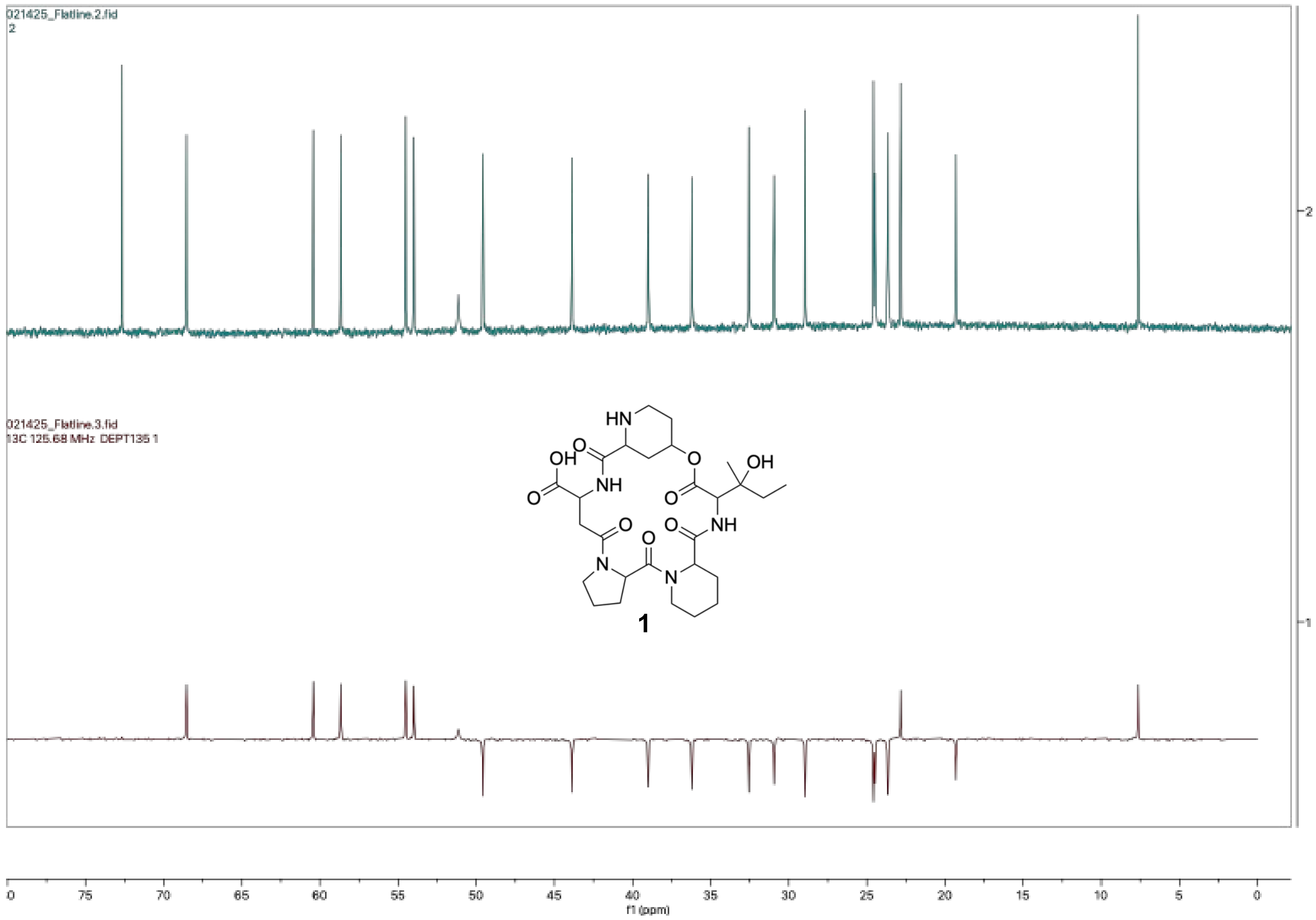

Figure S14. DEPT-135 NMR spectrum of **1** in D_2O (125 MHz).

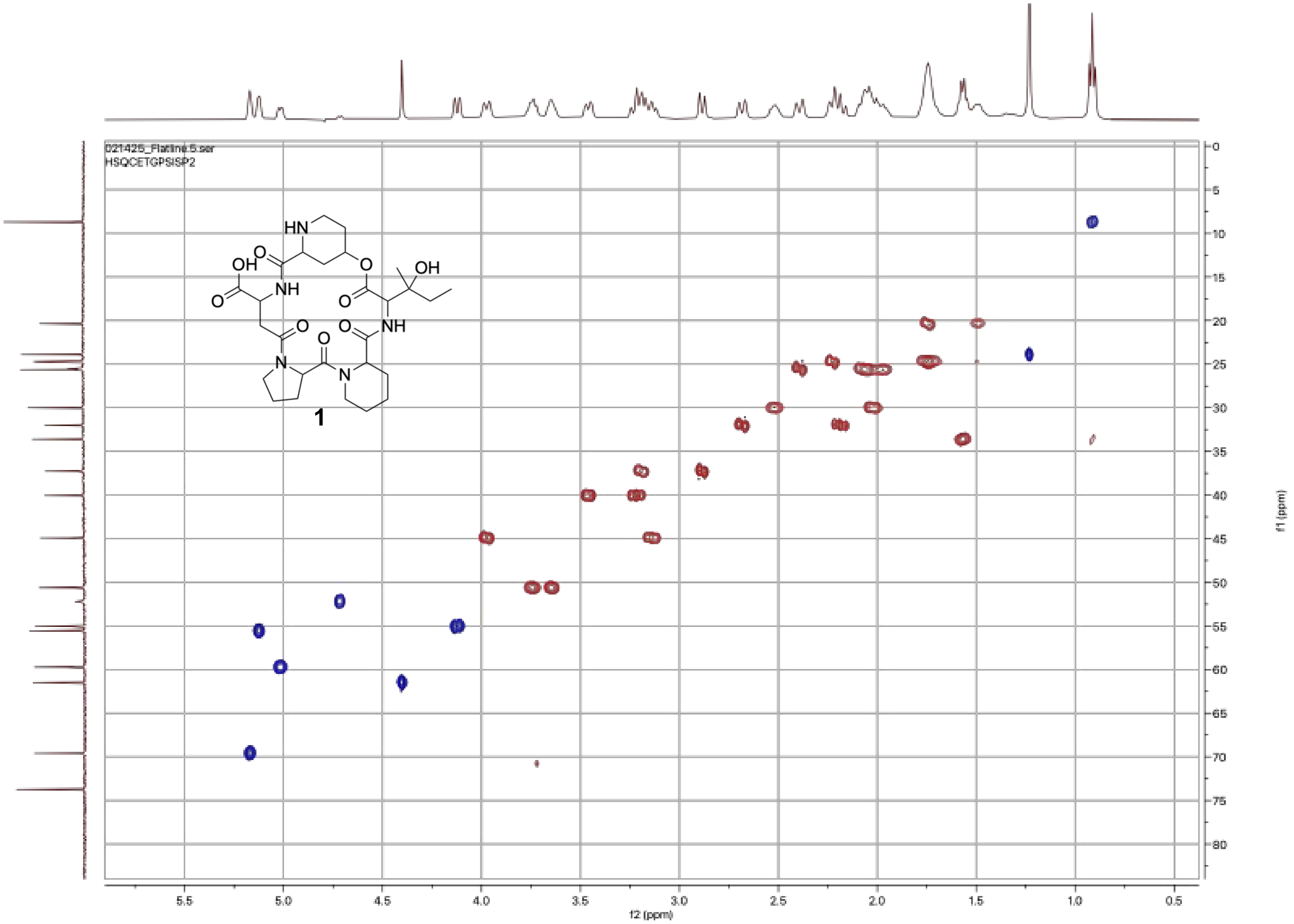

Figure S15. HSQC NMR spectrum of **1** in D_2O (^1H: 500 MHz, ^{13}C: 125 MHz).

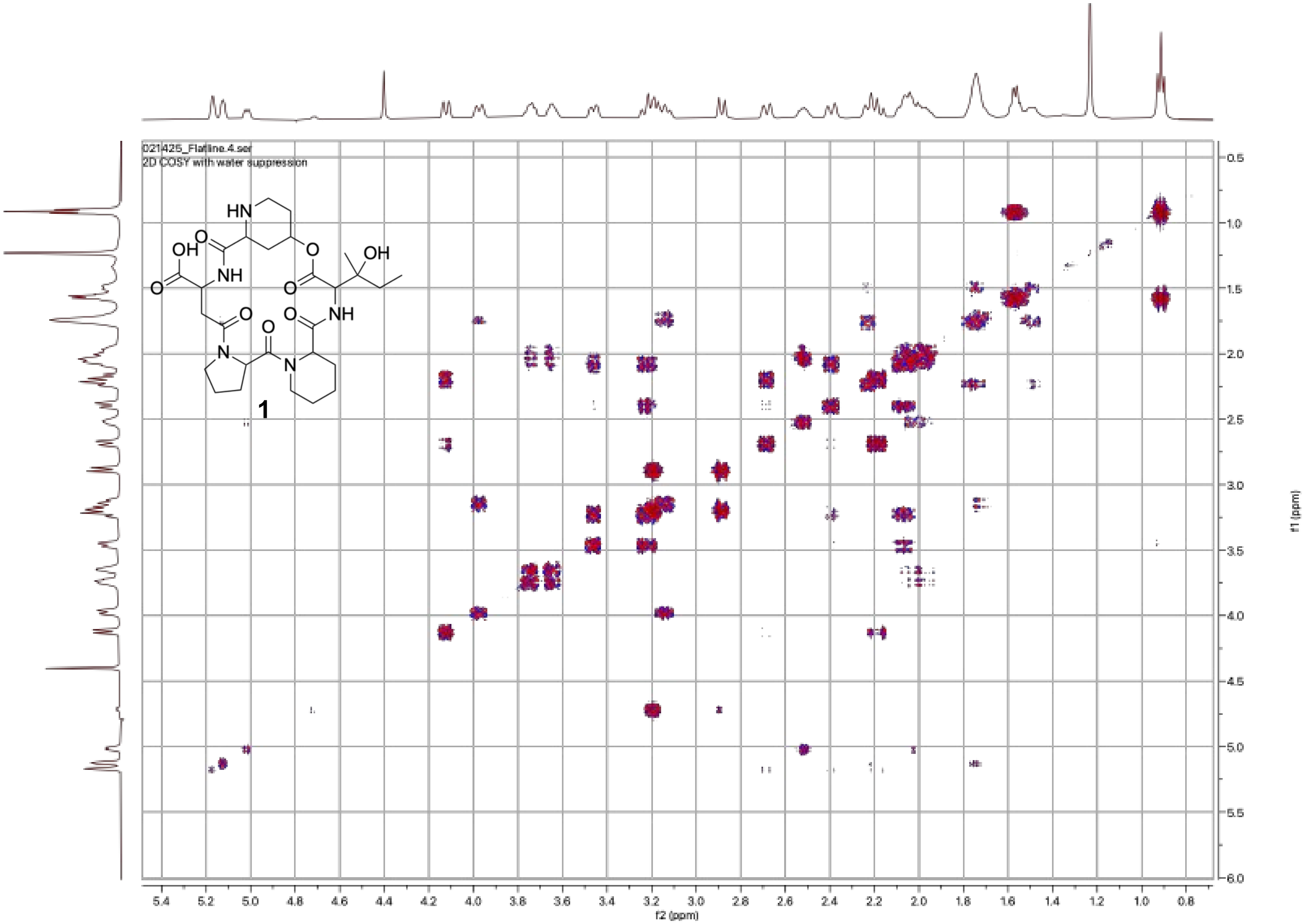

Figure S16. COSY NMR spectrum of **1** in D_2O (^1H: 500 MHz, ^{13}C: 125 MHz).

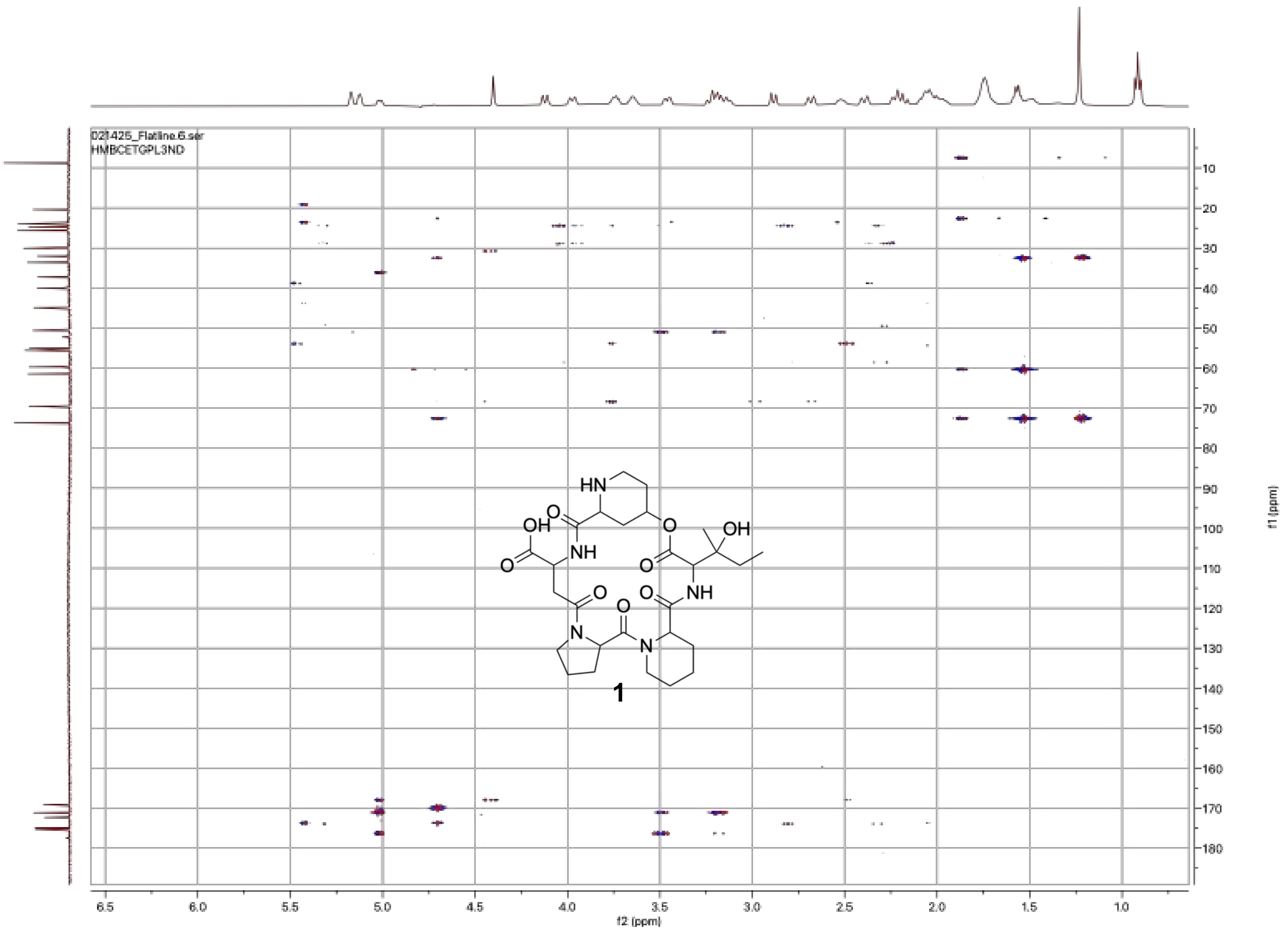

Figure S17. HMBC NMR spectrum of **1** in D_2O (^1H: 500 MHz, ^{13}C: 125 MHz).

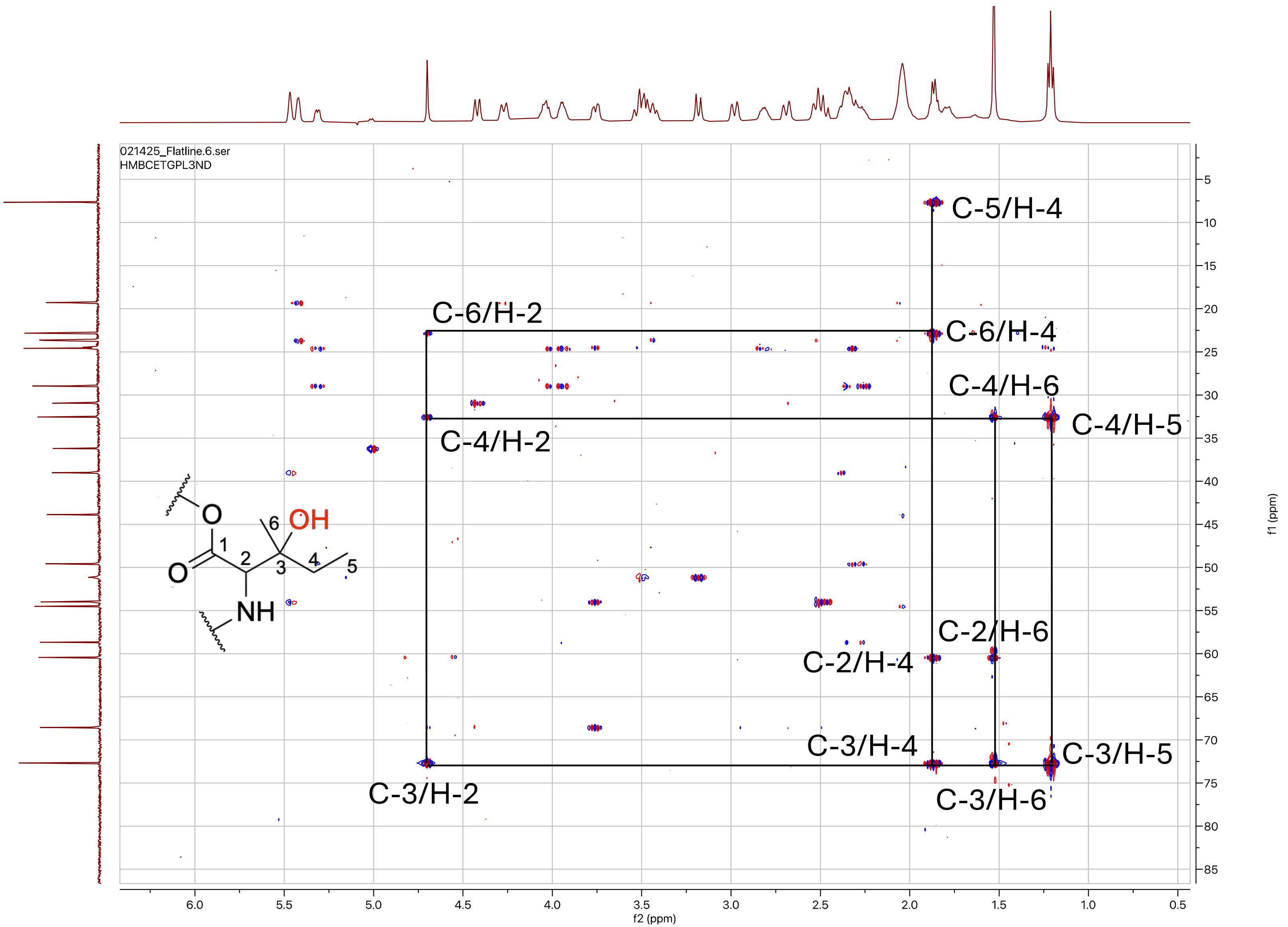

Figure S18. Spin system of hydroxy-Ile of **1** based on HMBC NMR correlations in D_2O (^1H: 500 MHz, ^{13}C: 125 MHz).

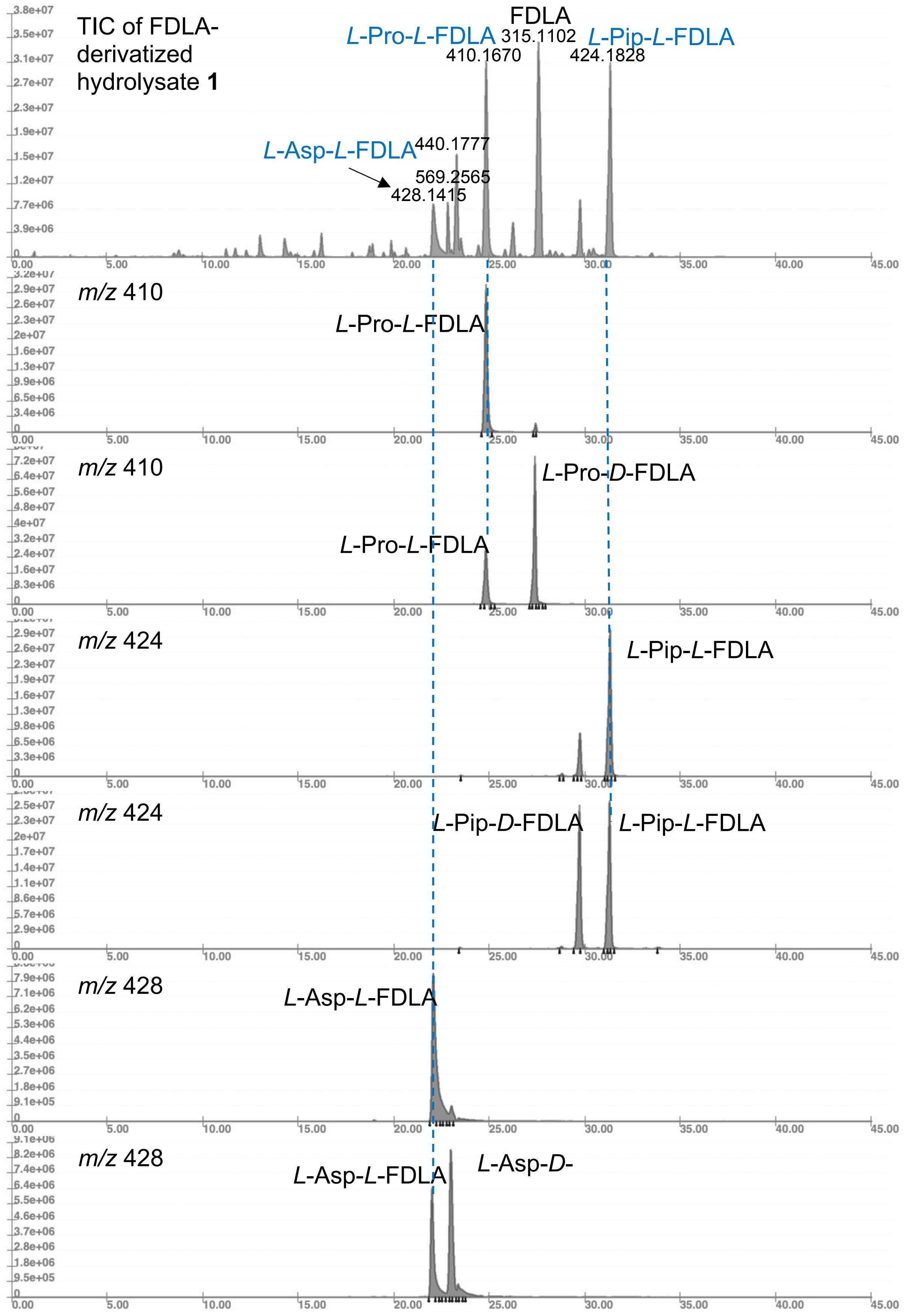

Figure S19. TIC of advanced Marfey's analysis of **1** compared to amino acid standards derivatized with *L*-FDLA or *D,L*-FLDA.

Table S4. Retention times of FDLA derivatives of amino acids derived from **1** and standards.

Residue	$[M+H]^+$	Authentic *L,L* *m/z* retention time	Authentic *D,L* *m/z* retention time	Observed *m/z* retention time of 1	Supposed configuration of 1
**Pro + FDLA**	410.17	24.80	27.39	24.82	*L*-Pro
**Pip + FDLA**	424.18	31.29	29.72	31.30	*L*-Pip
**Asp + FDLA**	428.14	22.03	23.00	22.05	*L*-Asp

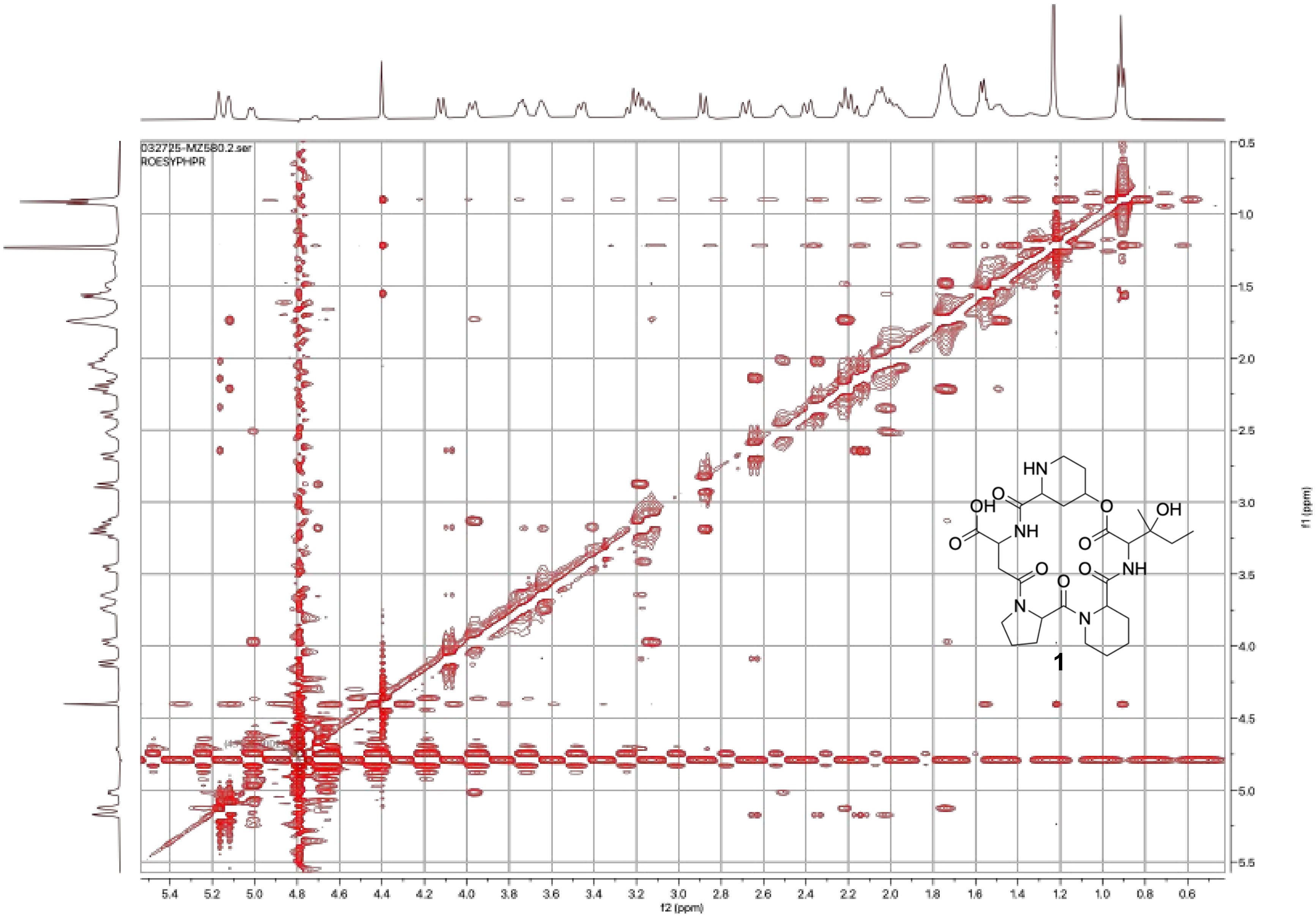

Figure S20. ROESY NMR spectrum of **1** in D_2O (500 MHz).

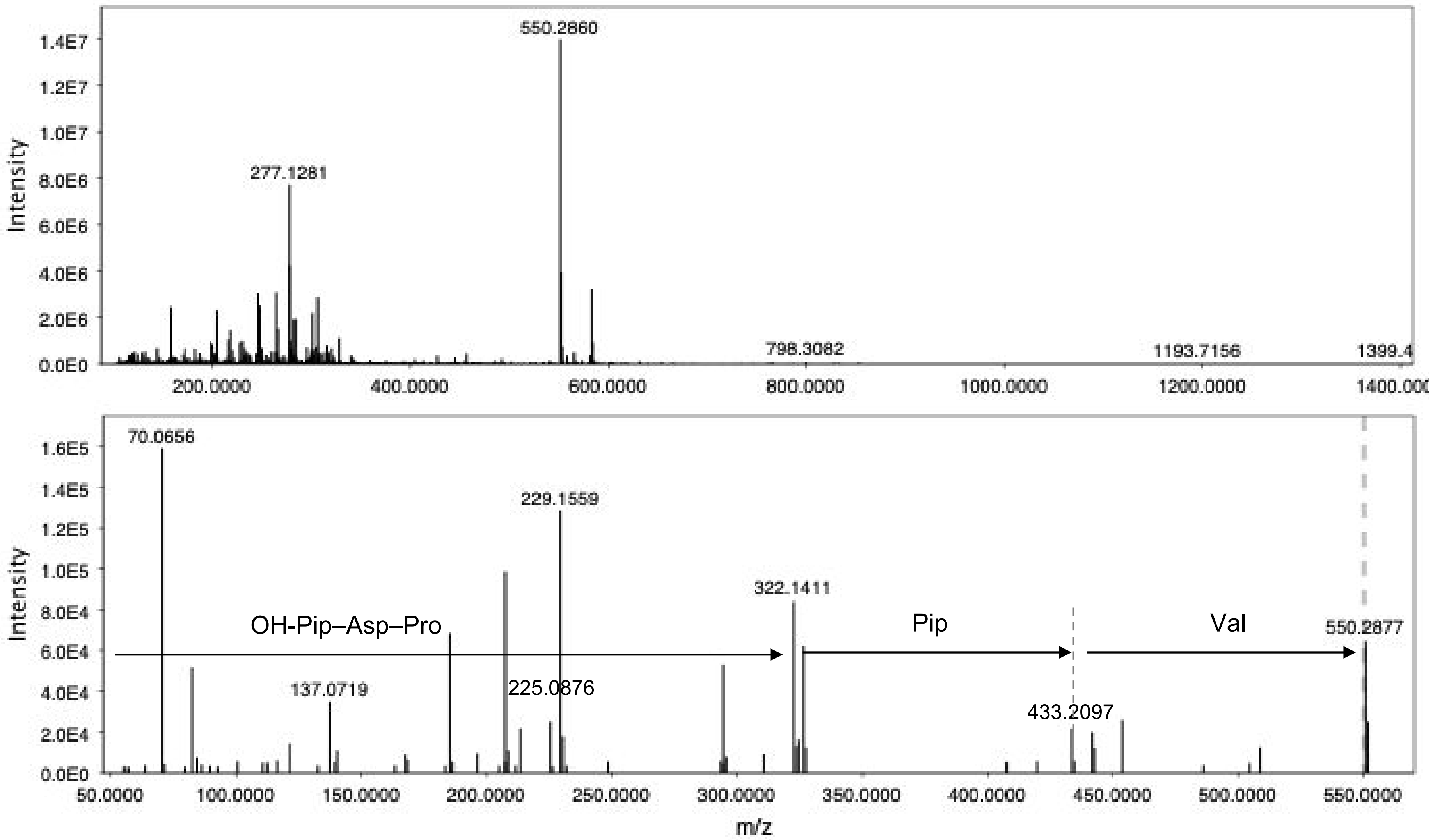

Figure S21. HR-ESI-MS and HR-ESI-MS/MS spectra of **2**.

A) TJLE1.1 (Δ*ctnA*)

BamHI-HF digestion

ctnA 5' and 3' flanks as probes

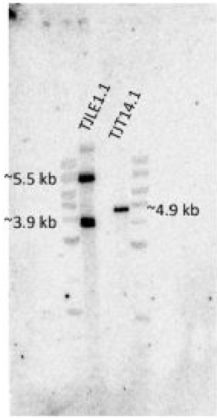

B) MJLE1 (TJLE1.1 HygR excision)

Expected product w/o HygR marker = 3265 bp

Expected product w/ HygR marker = 7811 bp

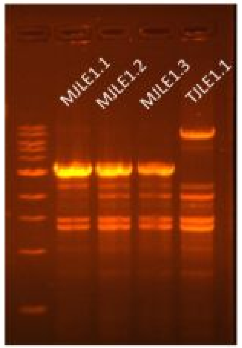

C) TJLE4 (Δ*ctnA* Δ*patL*)

BamHI-HF digestion

patL 5' and 3' flanks as probes

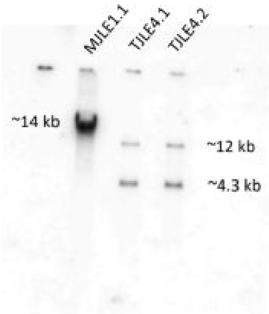

D) MJLE2 (TJLE4.1 HygR excision)

Expected product w/o HygR marker = 3130 bp

Expected product w/ HygR marker = 7772 bp

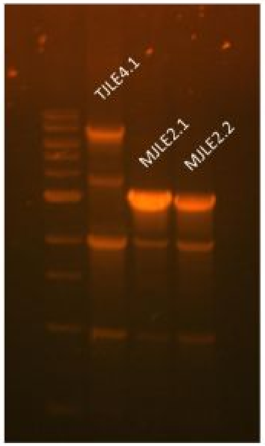

E)

TJLE16 (Δ*ctnA* Δ*patL* Δ*citS*)

MluI-HF digestion

citS 5' and 3' flanks as probes

Other lanes are Δ*citS* in other backgrounds

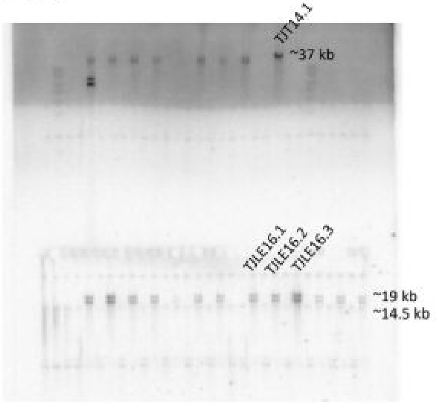

F)

MJLE3 (TJLE16.1 HygR excision)

Expected product w/o HygR marker = 3169 bp

Expected product w/ HygR marker = 7811 bp

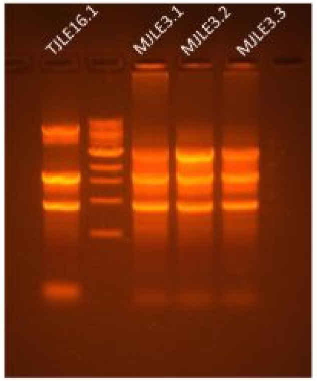

G)

TJLE17 (Δ*ctnA* Δ*patL* Δ*citS* Δ*roqA*)

NheI-HF digestion

roqA 5' and 3' flanks as probes

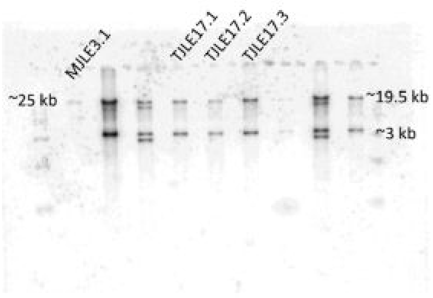

H)

MJLE4 (TJLE17.1 HygR excision)

Expected product w/o HygR marker = 3164 bp

Expected product w/ HygR marker = 7806 bp

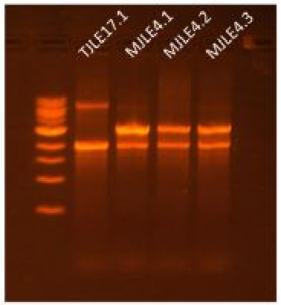

I)

TJLE27 (Δ*ctnA* Δ*patL* Δ*citS* Δ*roqA* Δ*cns*BGC)

MfeI-HF digestion

cns BGC 5′ and 3′ flanks as probes

*Chose an enzyme that cut within *cns* BGC and not the HygR marker, which should have yielded a ~5 kb product in parental. The other cut site was unknown due to genome sequence contig ending.

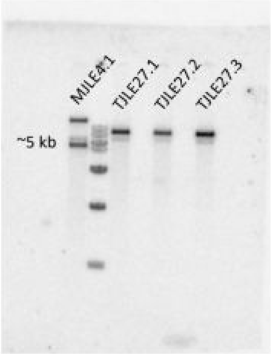

J)

MJLE5 (TJLE27.1 HygR excision)

Expected product w/o HygR marker = 4034 bp

Expected product w/ HygR marker = 8676 bp

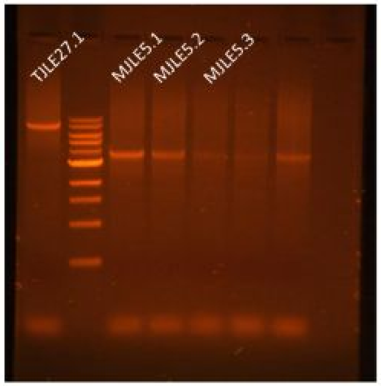

K)

TJLE30 (Δ*ctnA* Δ*patL* Δ*citS* Δ*roqA* Δ*cns*BGC ΔPEX2_030390)

SpeI-HF digestion

cheA 5′ and 3′ flanks as probes

*I missed an additional cut site in the 3′ region that is included in the parental

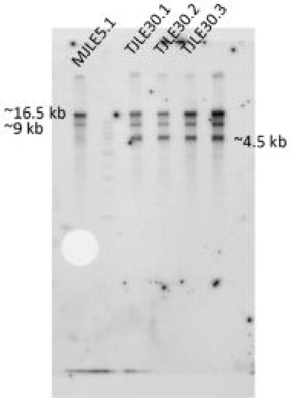

L)

MJLE6 (TJLE30.1 HygR excision)

Expected product w/o HygR marker = 3193 bp

Expected product w/ HygR marker = 7835 bp

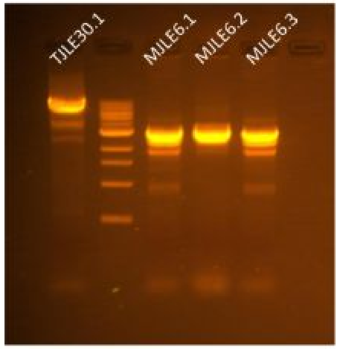

M) TJLE34 (Δ*ctnA* Δ*patL* Δ*citS* Δ*roqA* Δ*cns*BGC ΔPEX2_030390 Δ*adrD*)

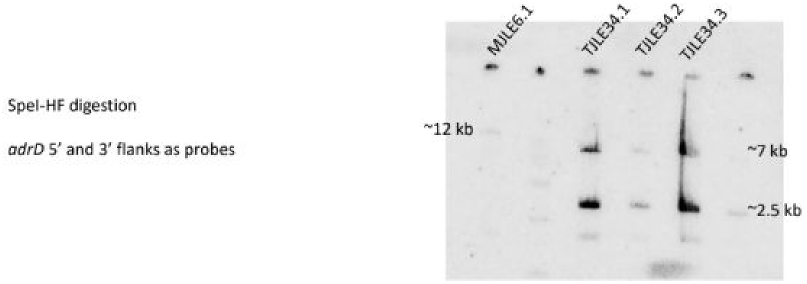

N) TBJH2.1 (Δ*ctnA* Δ*patL* Δ*citS* Δ*roqA* Δ*cnsBGC* Δ*PEX2_030390* Δ*PEXP_085540*): 5' side amplified, 3' side did not. We selected colonies 1.2, 3.1, 3.3, 5.1, and 6.1 and went directly to plating on rice media to check for compound rather than troubleshoot PCR.

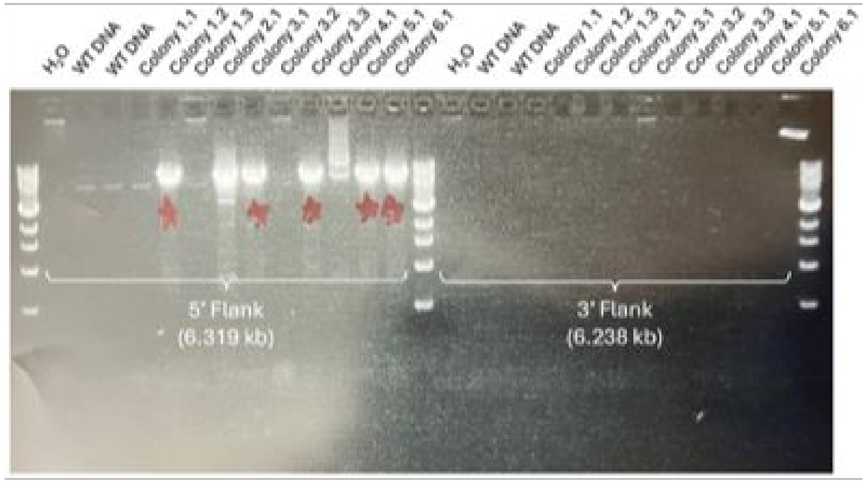

O) TBJH3.1 (Δ*ctnA* Δ*patL* Δ*citS* Δ*roqA* Δ*cnsBGC* Δ*PEX2_030390* Δ*mbjA*)

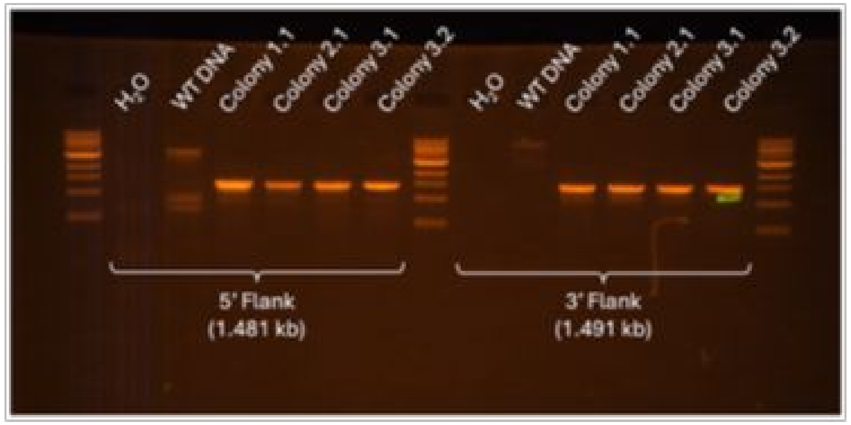

Figure S22. Southern blot and PCR confirmations of all deletion strains in this study.

Table S6. Primer sequences designed in this study.

Primer Name	Sequence
PEXP_85540_5'_Flank_Fwd	tactacgcaacggttgcag
PEXP_085540_5'_Nested_Fwd	cagagattcgccctgcg
PEXP_085540_5'_Flank_Rev	ACCTATAGGACCTGAGTGATGCGctcgtataggagccgtggag
PEXP_085540_3'_Flank_Fwd	GCATAATATGGTCCATCTAGTGCGCAatgtggctctgcattcatttgc
PEXP_085540_3'_Nested_Rev	ttggcactcctatgggacc
PEXP_085540_3'_Flank_Rev	tggtccatgaccttggaccc
MbjA_5'_Flank_Fwd	cagagtgtctccttacggctg
MbjA _5'_Nested_Fwd	gcagatggcccgcataga
MbjA _5'_Flank_Rev	ACCTATAGGACCTGAGTGATGCGgcaaatttcgatgtcatcaagttcataag
MbjA _3'_Flank_Fwd	GCATAATATGGTCCATCTAGTGCGCAgacctgttattgatcggaagggt
MbjA _3'_Nested_Rev	gcggatcttgttccgatcc
MbjA _3'_Flank_Rev	cggaacaaacactccgctac

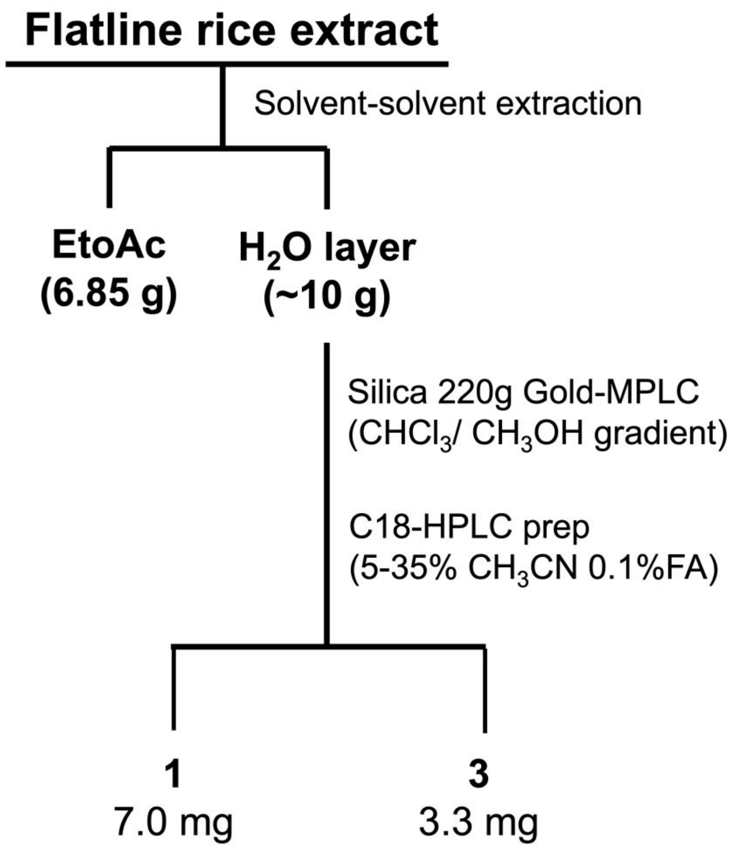

Figure S23. Isolation chart of cyclopeptides **1** and **MBJ-0110 (3)**.

References

(1) Shimizu, K.; Keller, N. P. Genetic Involvement of a cAMP-Dependent Protein Kinase in a G Protein Signaling Pathway Regulating Morphological and Chemical Transitions in Aspergillus Nidulans. *Genetics* **2001**, *157* (2), 591–600.
(2) Atlas, R. M. *Handbook of Microbiological Media*; CRC press, 2004.
(3) Greene Andrew V.; Keller Nancy; Haas Hubertus; Bell-Pedersen Deborah. A Circadian Oscillator in Aspergillus Spp. Regulates Daily Development and Gene Expression. *Eukaryotic Cell* **2003**, *2* (2), 231–237. https://doi.org/10.1128/ec.2.2.231-237.2003.
(4) Fountain, J. C.; Bajaj, P.; Pandey, M.; Nayak, S. N.; Yang, L.; Kumar, V.; Jayale, A. S.; Chitikineni, A.; Zhuang, W.; Scully, B. T.; Lee, R. D.; Kemerait, R. C.; Varshney, R. K.; Guo, B. Oxidative Stress and Carbon Metabolism Influence Aspergillus Flavus Transcriptome Composition and Secondary Metabolite Production. *Scientific Reports* **2016**, *6* (1), 38747. https://doi.org/10.1038/srep38747.
(5) Eagan, J. L.; Digman, E. R.; den Boon, M.; Regalado, R.; Rawa, M. S. A.; Hull, C. M.; Keller, N. P. Patulin Inhibition of Specific Apple Microbiome Members Uncovers Hanseniaspora Uvarum as a Potential Biocontrol Agent. *Phytopathology®* **2025**, *115* (2), 117–127. https://doi.org/10.1094/PHYTO-06-24-0189-R.
(6) Kawahara, T.; Itoh, M.; Kozone, I.; Izumikawa, M.; Sakata, N.; Tsuchida, T.; Shin-ya, K. MBJ-0110, a Novel Cyclopeptide Isolated from the Fungus Penicillium Sp. F25267. *The Journal of Antibiotics* **2016**, *69* (1), 66–68. https://doi.org/10.1038/ja.2015.78.
